# Limits to the Rate of Adaptive Substitution in Sexual Populations

**DOI:** 10.1371/journal.pgen.1002740

**Published:** 2012-06-07

**Authors:** Daniel B. Weissman, Nicholas H. Barton

**Affiliations:** Institute of Science and Technology Austria, Klosterneuburg, Austria; University of Oxford, United Kingdom

## Abstract

In large populations, many beneficial mutations may be simultaneously available and may compete with one another, slowing adaptation. By finding the probability of fixation of a favorable allele in a simple model of a haploid sexual population, we find limits to the rate of adaptive substitution, 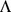, that depend on simple parameter combinations. When variance in fitness is low and linkage is loose, the baseline rate of substitution is 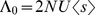, where 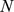 is the population size, 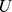 is the rate of beneficial mutations per genome, and 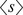 is their mean selective advantage. Heritable variance 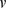 in log fitness due to unlinked loci reduces 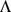 by 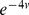 under polygamy and 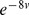 under monogamy. With a linear genetic map of length 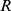 Morgans, interference is yet stronger. We use a scaling argument to show that the density of adaptive substitutions depends on 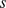, 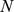, 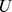, and 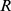 only through the baseline density: 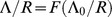. Under the approximation that the interference due to different sweeps adds up, we show that 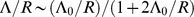, implying that interference prevents the rate of adaptive substitution from exceeding one per centimorgan per 200 generations. Simulations and numerical calculations confirm the scaling argument and confirm the additive approximation for 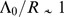; for higher 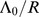, the rate of adaptation grows above 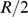, but only very slowly. We also consider the effect of sweeps on neutral diversity and show that, while even occasional sweeps can greatly reduce neutral diversity, this effect saturates as sweeps become more common—diversity can be maintained even in populations experiencing very strong interference. Our results indicate that for some organisms the rate of adaptive substitution may be primarily recombination-limited, depending only weakly on the mutation supply and the strength of selection.

## Author Summary

In small populations, adaptation may be limited by a lack of beneficial alleles on which selection can act; in such populations, increasing the supply of mutations (by increasing the population size or the rate of beneficial mutation per individual) proportionally increases the rate of adaptation. However, when multiple beneficial mutations arise simultaneously, they will typically occur in different individuals and will compete against each other, slowing adaptation. Recombination (sex) alleviates this interference among mutations by bringing them together in the same individuals. By analyzing and simulating a simple model of an adapting sexual population, we find that interference prevents the rate of adaptive substitutions from greatly exceeding one substitution per centimorgan in every 200 generations. Populations with infrequent outcrossing, such as many microbes and plants, may approach this limit. In these populations, the rate of adaptive substitutions is hardly affected by increasing the mutation supply or the strength of selection, but grows proportionally (up to very high rates) as recombination increases.

## Introduction

In an adapting population, beneficial alleles may be spreading simultaneously at multiple genetic loci. New beneficial mutations usually arise in different individuals, and thus compete with each other for fixation [1], [2]. In asexual populations, this “clonal interference” among alleles can drastically reduce the rate of adaptation [3]–[11]. In sexual populations, recombination can speed adaptation by breaking up negative associations among beneficial alleles [1], [2]. While this effect is implied by Weismann's explanation for the advantage of sex [12], and was first investigated mathematically nearly half a century ago [13]–[16], there has been surprisingly little explicit treatment of the effects of interference on rates of adaptation. This is largely because the substantial body of theory on the evolution of recombination has focussed on the fate of modifiers of recombination, and on the effects of deleterious rather than favorable mutations (e.g. [17]–[20]; reviewed by [21]). The effect on the rate of adaptation itself has remained implicit. Recently, there has been intense interest in adaptation by asexual populations, stimulated by laboratory selection experiments on bacteria, and this has led on to theoretical studies of multilocus evolution in sexual populations [22]–[30], although these have generally focused on unlinked loci in facultative sexuals.

While not much is known quantitatively about the effect of interference among beneficial mutations in sexual populations, it is plausible that it is significant. Evidence of clonal interference has been repeatedly observed in experimental evolution of viruses [31]–[35], bacteria [36]–[40], and eukaryotic microbes [6], [41]–[44], and selected polymorphisms at linked loci must occur simultaneously in plants and animals undergoing artificial selection – the motivation for Hill and Robertson's initial analysis [14]. Thus, it is important both to understand how linkage among beneficial alleles affects adaptation, and how it can be detected in natural populations from sequence data.

A simple way to measure adaptation is by the accumulation of favorable mutations. The rate of accumulation, 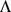, is equal to the product of the number of haploid individuals, 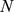, the beneficial mutation rate per genome per generation, 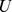, and the average probability that a single new mutation will ultimately fix, 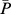: 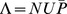. (See Table 1 for a summary of the notation.) 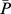 itself will in turn generally depend on 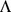, because each mutation that sweeps to fixation will reduce the chance that other mutations will fix. (This reduction in fixation probability is an example of the Hill-Robertson effect [45]). To see why this is so, note that all pre-existing beneficial alleles that are not present in the original mutant individual must be lost in the absence of recombination, as must all new mutations that occur on the ancestral background [1], [2]. Copies of other alleles that are in individuals carrying the sweeping allele will have an increased fixation probability, but because this increase is on average far less than the decrease in fixation probability for copies on the ancestral background, the net effect of the sweep is negative. The fixation probability thus decreases as the rate of sweeps increases.

**Table 1:** Symbol definitions.

Symbol	Definition
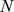	Haploid population size
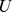	Genomic beneficial mutation rate
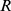	Total genetic map length
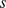	Selective advantage of beneficial mutations
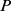	Probability of fixation of a beneficial mutation
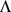	Genomic rate of fixation of beneficial mutations
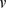	Heritable variance in log fitness in the population
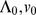	Values of 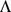 and 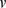 in the absence of interference
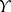	Expected time for a pair of neutral lineages to coalesce

The definitions of the main symbols used in the text. 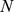, 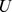, 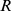, 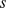, 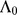, and 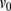 are population parameters, and 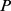, 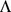, 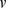, and 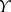 are variables. In addition, we use 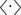 to denote the expectation of a variable taken over a distribution of selective coefficients 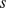, and 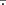 to denote the expectation over possible genetic backgrounds.

Here we derive simple approximate expressions for 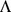 by analyzing a basic model of an adapting population. We begin by considering unlinked loci, but then focus on the recombination model of most biological interest, namely a linear genome with cross-overs randomly scattered at a total rate 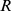 per generation. We use a robust scaling argument to show that the proportional reduction to 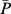 caused by interference depends only on the density of sweeps, 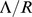. We derive an explicit form for 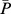 as a function of 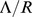, under the approximation that the effects of multiple sweeps are additive. We find that, in sufficiently large populations, 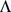 is proportional to 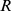 but nearly independent of the rate at which beneficial mutations are produced (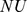), indicating that adaptation is primarily limited by the rate at which recombination can bring beneficial alleles together. (A preliminary version of these results was outlined by [46].) Simulations confirm the scaling argument, and show that the expression for 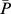 is accurate up to 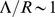. Finally, we consider the effect of multiple sweeps on neutral diversity, and find that it scales differently than the effect on adaptation: neutral diversity can be greatly reduced even when sweeps are too sparse to interfere with each other, but it is not much more reduced when interference is strong.

## Results

### The model

We consider a well-mixed population of 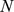 haploid individuals. All mutations are beneficial and the effects of different alleles on fitness multiply. There is a constant genomic beneficial mutation rate 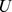, regardless of genetic background, so that beneficial mutations are never exhausted. Our model can thus be seen as a best-case scenario for adaptation, ignoring the deleterious mutations, negative epistasis among beneficial mutations, and lack of available beneficial mutations that presumably limit adaptation in many real populations. (We consider the effect of deleterious mutations and population structure in the Discussion.) Under these assumptions, the population will approach an expected steady long-term rate of substitution, 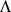; we focus on populations close to this steady state. (We discuss fluctuations in the rate of substitution in Text S5 and Figure S9.)

### Background: Fixation probabilities and adaptation in the absence of interference

In the absence of interference from linked alleles, a single allele with advantage 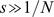 has probability 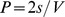 of going to fixation, where 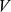 is the variance in offspring number among individuals [47], [48]. (This expression also applies to more complicated demographic models, with 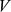 taken to be the variance in reproductive value [49].) For the rest of this paper, we will assume that individuals' offspring distributions are approximately Poisson, corresponding to a base value (in the absence of interference) of 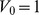, as under the Wright-Fisher model (Eq. 1.48 of [50]). The expected probability of fixation of a beneficial mutation is therefore 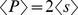 and the baseline rate of accumulation of favorable alleles is 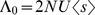. (We use 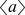 to indicate the expectation of quantity 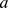 over possible values of 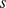, and 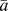 to indicate the expectation over individuals in a population; for the baseline rate 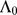 we are neglecting variation in the genetic backgrounds among individuals.)

It will be helpful to consider log fitness; for an individual with 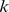 favorable alleles, each providing advantage 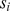, this is 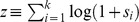. By Fisher's “Fundamental Theorem” [1], the rate of increase of the population mean log fitness, 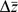, is given by the heritable variance in log fitness, 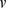. (Here we are neglecting the direct effect of new mutations, which we address below.) A substituted allele with advantage 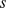 makes a contribution 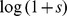 to 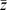, so the rate of increase is 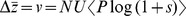. In the absence of interference, the baseline rate of increase is 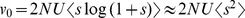 (for 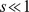).

### Complete recombination

We begin by assuming that in each (discrete) generation, each individual is generated by choosing its genes independently from a common pool (“complete recombination”). Thus, the state of each gene is statistically independent of the other genes, or in other words, there is no linkage disequilibrium. This does not correspond to any real organism, but could be realized in principle: it corresponds to a kind of mass meiosis, in which all members of the population take part. (This procedure can be approximated by multiple rounds of random mating with no selection, and is used directly in some genetic algorithms [51].)

Since each individual chooses all of its alleles independently, its log fitness is the sum of independent contributions from all the polymorphic loci. When many ongoing selective sweeps contribute to variance in fitness, 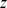 will be approximately normally distributed (with variance 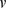). In this case, the variance in the number of offspring of a new allele, taken over all genetic backgrounds, is 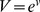. The fixation probability of an allele with advantage 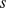 is therefore reduced to 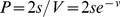. Thus, the net rate of increase in mean log fitness, 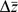, is reduced by a factor 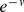, and so we have 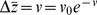. This can be rewritten as 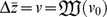, where 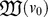 is the product log function (also known as the Lambert W function), which is approximately 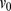 for 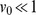, and 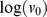 for 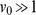. Thus, if the rate of adaptation is so extremely high that most variance in offspring number is due to selective sweeps (rather than simple drift), the rate of adaptation only increases very slowly (logarithmically) with the number of new mutations entering the population.

In deriving this formula, we have assumed that there are enough selective sweeps that 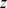 is approximately normally distributed. We have checked this approximation by simulating the full model, and find very close agreement over a wide range of parameters. (See Text S1 and Figure S1.)

### Unlinked loci

We now extend this argument to a more realistic model, and find the same qualitative result. We consider a Wright-Fisher population, in which each individual is the offspring of two parents in the previous generation, chosen with probability proportional to their fitnesses. We assume the infinitesimal model, under which two parents with trait values 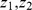 produce offspring with values normally distributed around the mid-parent value 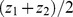, and variance 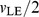, where 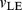 is the variance of 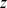 in a population at linkage equilibrium [52]. This model has been found to be a good approximation for the response to selection of many quantitative traits in sexual populations [53]. Under the assumption of weak selection per locus, and free recombination (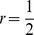), linkage disequilibria among alleles sweeping to fixation are negligible, and so 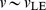. (Note, however, that linkage disequilibrium decays only at a rate 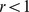 per generation, distinguishing this model from the complete recombination model above.)

We can consider two models: polygamous and monogamous. In the first, an individual with trait value 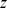 has a Poisson number of offspring with expectation proportional to 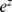. Each offspring is produced with a different mate, with an individual with trait value 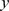 chosen as a mate with probability proportional to 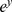. In the second, pairs with trait values 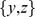 form at random, and produce a Poisson number of offspring, with expectation proportional to 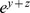. Because all of an individual's offspring are influenced by the same mate, this model introduces substantially more random drift. In Text S2 , we show that in both models, fixation probability of a new mutation is proportional to the square of the fitness of the individual in which it arises (i.e., 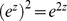). With polygamy, the average fixation probability is reduced by a factor 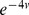. Arguing as before, we find that the overall rate of adaptation is given by
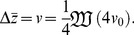
(1)


This is consistent with Robertson's heuristic argument that variation in fitness that is inherited with probability (i.e., recombination fraction) 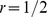 has 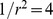 times the effect of non-inherited fitness variation [54]. However, with monogamy, inherited variation in fitness has an even larger effect, reducing fixation probability by a factor 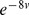, and giving a rate of adaptation 
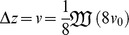
. (Note that the preliminary expression in [46] is incorrect.) We have checked Eq. (1) by direct simulation of the infinitesimal model (Text S2 and Figure S2). It is straightforward to extend this result to populations of facultative sexuals that outcross at regular intervals; in this case a “generation” should be seen as the several rounds of clonal reproduction between outcrossing events, with all selective coefficients scaled up accordingly. [26], [27] have recently modeled a different kind of facultative sex; see the Discussion for a comparison of our results.

### A linear map

We now turn to the case of most biological interest, namely, loci arranged linearly on chromosomes, with recombination within chromosomes occurring via crossovers. When there are many chromosomes or each chromosome is long (so that the total genetic map length 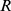 is 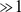), most loci will be effectively unlinked (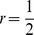), and so we expect these to reduce fixation probability by a factor 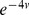, assuming polygamy. However, tightly linked loci are expected to make a substantial contribution. Since, according to a straightforward generalization of [54], those at map distance 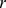 are expected to reduce fixation probability by 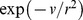, the average over a linear map should diverge as 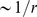 for small 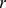. Plainly, a more sophisticated argument is needed to deal with tightly linked loci.

In general, we must follow the fixation probability of an allele, considered as a function of the genetic background 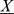 in which it sits; the vector 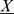 is a binary string which represents the 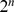 genotypes that are possible with 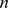 concurrent sweeps. When recombination and selection occur at rates small compared to the generation time but large compared to the mutation rate, the fixation probability of an allele conferring advantage 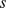 on a genetic background 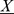 evolves according to:
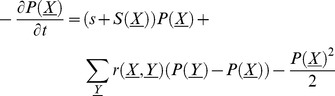
(2)(from Eq. 4 of [55]). Here 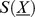 is the net selective advantage of background genotype 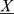, relative to the population mean. 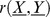 is the rate at which a focal allele on background 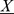 recombines onto background 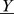; this depends on both recombination rates and genotype frequencies, 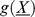, which will vary in time. (Intuitively, in the right-hand side of Eq. (2) , the first term describes the increase or decrease in the allele frequency due to selection, the second term describes how recombination shuffles the allele's genetic background, and the third term describes the effect of drift.)

The quantity of most interest is the average fixation probability over all possible genetic backgrounds, 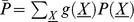. If we take the time derivative of this average probability, we find that terms in Eq. (2) due to selection on the background, and recombination, cancel, giving:
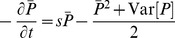
(3)(Text S3). Fixation probability is always reduced below 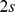 by variation in fixation probability across backgrounds (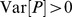). In the special case where 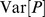 is constant through time, we have 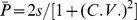 where 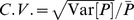. Factors that increase the short-term rate of drift in a way that does not depend on genetic background (unequal sex ratio, uncorrelated fitness variance, etc.) can be included by multiplying the last term in Eq. (2) by a factor 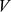, the variance in reproductive value. This result is remarkably general: it does not depend on the pattern of recombination, and it does not assume additive effects: 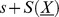 is simply the net rate of increase of the focal allele when on genetic background 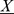. (The effect of the focal allele, 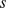, must be additive, but remaining alleles can have arbitrary epistatic interactions with each other, as described by 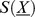.) However, Eq. (3) does not help us calculate the magnitude of the reduction in the fixation probability, since 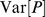 depends on recombination, selection, and the background genotype frequencies (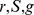).

Note that the derivation of Eq. (3) still holds when we extend the genetic background and recombination to include spatial location and migration in a structured population. If an allele has the same selective advantage, 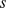, everywhere, then the fixation probability is equal to 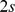, independent of structure [56], [57]. If selection varies from place to place, with mean 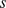, then Eq. (3) shows that the average fixation probability is necessarily reduced below 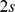. In this context, Eq. (3) may be related to a similar expression found by [58]; the possible connection is discussed in Text S6.

### The net reduction in the rate of adaptation depends only on the baseline density of sweeps, 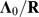


When there are many possible genetic backgrounds due to multiple interfering sweeps, it is generally difficult to calculate 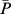 exactly from Eqs. (2) and (3). In the following, we derive an approximate expression for 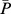 that is accurate up to very strong interference. For simplicity, we will assume in this section that all mutations confer the same selective advantage, 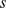, regardless of genetic background. (Our argument holds more generally as long as the distribution of selective effects has a characteristic scale 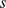; see below.) First, we use a scaling argument to show that in large populations, the rate of selective sweeps per unit map length, 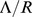 (which we refer to as the “density” of sweeps), depends on 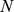, 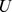, 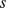, and 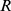 only through the rate in the absence of interference between loci, 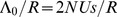. In other words, we show that there is a function 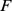 such that 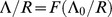. Later, we use simulations to confirm this argument, even for very strong interference.

**Figure 1:**
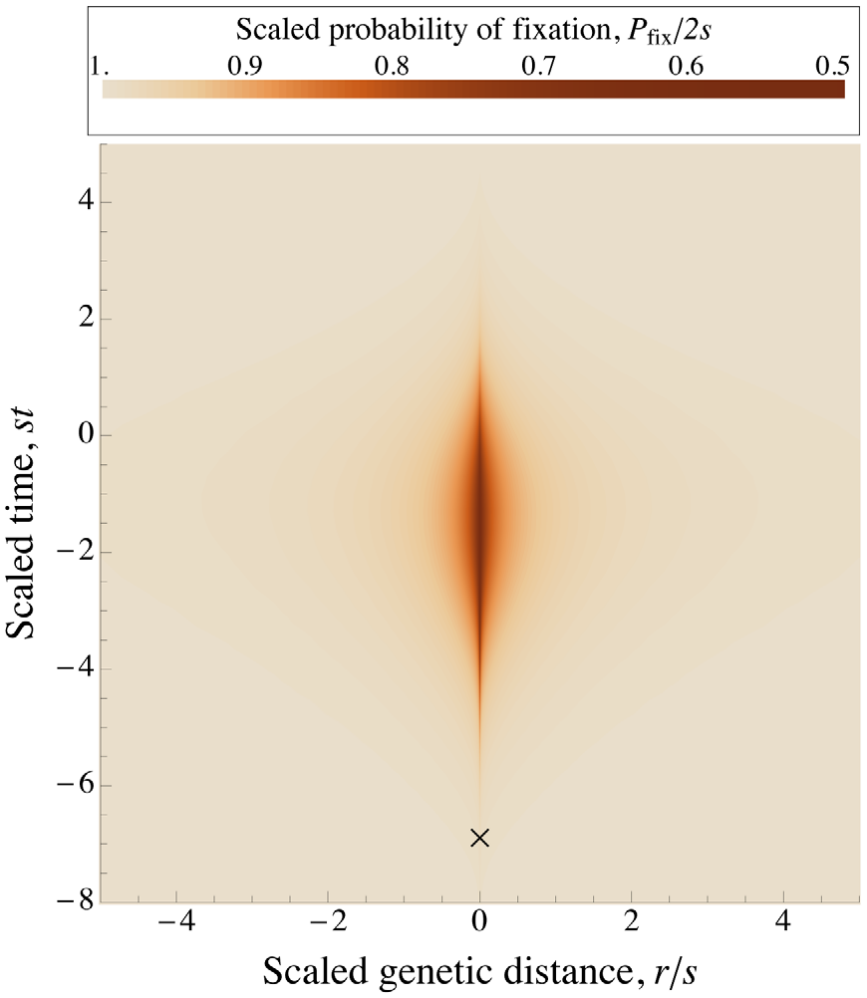
A selective sweep causes interference over a time 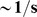 and a genetic distance 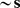. Fixation probability of a new mutation with advantage 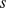 occurring after an interfering sweep with the same selective advantage 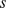. The fixation probability 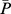, scaled by its baseline value 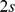, is plotted against the scaled map position of the new mutation relative to the interfering sweep, 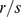, and its scaled time of occurrence relative to the time at which the interfering sweep reaches frequency 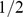, 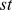. Note that the relationship between these scaled variables is independent of 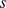, as long as 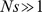. The X marks the time when the interfering sweep is at frequency 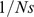 for 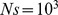; it is assumed to follow a deterministic trajectory. The sweep causes the most interference once it becomes common (frequency 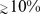), and causes little interference to common alleles (i.e., alleles that arise around the same time or earlier). 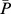 is calculated numerically using Eqs. (2) and (3) .

The key observation is that alleles are most vulnerable to interference when rare, but cause the most interference when moderately common. (Intuitively, a mutant allele causes the most interference when it is near frequency 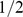 – frequent enough to significantly affect other alleles, but not so frequent that most other alleles are on the mutant background; see Figure 1 and Figure S3.) We assume that 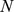 is very large, so that there is a number 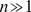, 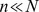 such that alleles which are present in 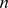 copies are established (i.e., are very likely increase to fixation along a roughly deterministic trajectory), while still being at low frequency in the population. This allows to us to make the crucial approximation that each mutation has a negligible effect on other mutations prior to its establishment, separating the roughly deterministic increase of alleles that are destined to fix (and which interfere with the fixation of others) from the stochastic fluctuations of rare alleles. For a given pattern of established sweeps, these rare alleles can be treated as independent branching processes, with fixation probability given by Eq. (2) . Notice that we can rescale Eq. (2) by writing it in terms of 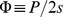, 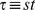, and 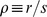, and letting 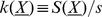 be the difference between the number of beneficial alleles in background 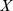 and the average number:
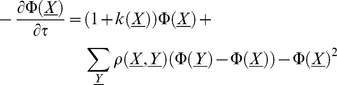
(4)This rescaled equation does not explicitly depend on 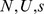, or 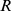 – only implicitly, through the dependence of 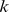 and 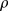 on the genotype frequencies, 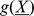. This is still true when we average over genotype frequencies to find the scaled version of Eq. (3) . Thus, the scaled probability of fixation of a new mutation that falls on a random genetic background, 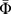, depends on 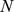, 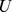, 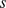, and 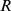 only through their effect on the number and pattern of interfering sweeps.

To find the dependence of 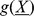 on the population parameters, we further assume that 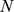 and 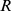 are large enough that, by the time a sweeping allele becomes common, any linkage disequilibrium with other common alleles will have decayed sufficiently that it can be neglected. (We revisit this assumption below.) In this case, we can approximate 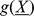 by the product of the frequencies of all the alleles in 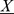, with each allele following a deterministic trajectory. When this is valid, the trajectories of common alleles are independent of 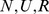, and 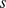 (when written as functions of the scaled time 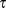). Thus, the parameters affect 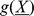 only through their effect on the distribution of sweeps in time and across the genome, and this distribution (in terms of the scaled time and scaled map distances) entirely describes their effect on 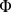.

**Figure 2:**
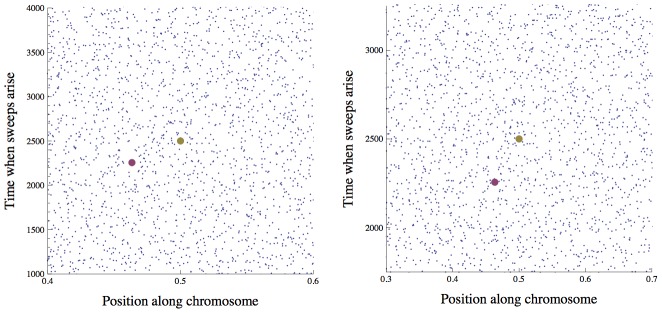
The distribution of sweeps in time across the genome. Points show the beginnings of simulated selective sweeps. The distribution over time and map length appears approximately uniform. Time is in generations from the beginning of the simulation, and position is map distance in Morgans from the end of the chromosome. In the right panel, the time scale is halved and the length scale is doubled compared to the left panel, illustrating the effect of a doubling of 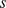 on the scaled distribution of sweeps that enters into Eq. (4) for the scaled probability of fixation 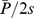. If we consider a focal mutation occurring in the middle of the chromosome at generation 2500 (the large gold dot), the rescaling changes the interference it experiences from any given sweep (e.g., the one marked by the large purple dot), but the total expected interference from the whole distribution of sweeps remains unchanged. Simulation parameters are chosen such that there is strong interference: 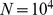, 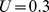, 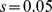, 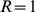.

We now make the final approximation that sweeps occur at approximately uniformly and independently distributed times and map positions, as they would in the absence of interference. In this case, the distribution, and therefore 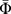, depends only on the density, 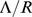. (The scaled and unscaled densities of sweeps are the same, since the scaling factors 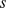 for time and 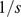 for map length cancel; see Figure 2.) There is a subtlety to this argument. If we consider a given set of sweeps, occurring at defined times and map positions, then their effects on a randomly placed mutation would depend on the strength of selection, and our scaling argument would fail. However, because the distribution of sweeps is invariant under rescaling, the fixation probability averaged over all possible configurations of sweeps is unchanged (Figure 2).

**Figure 3:**
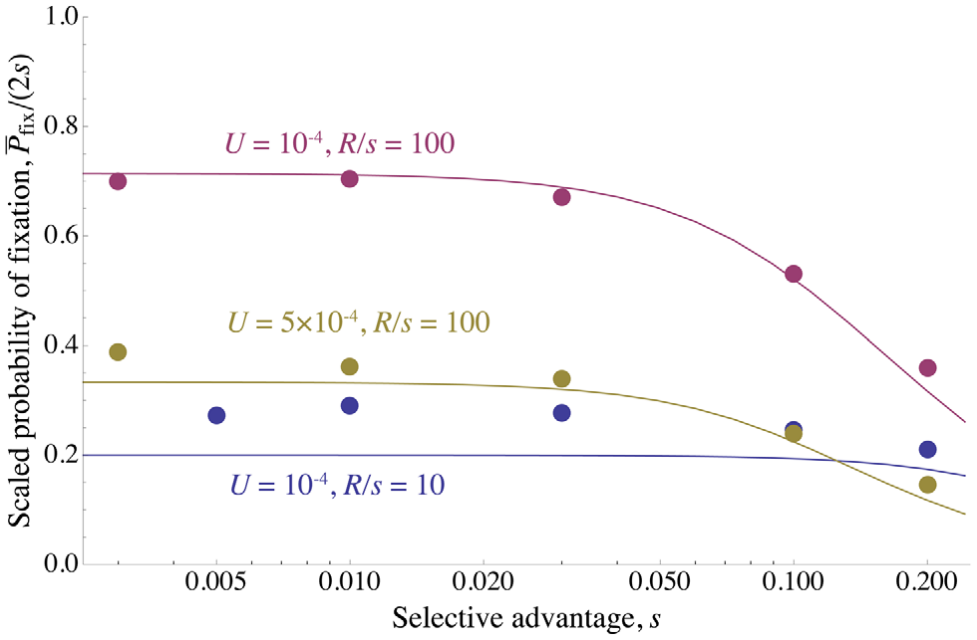
Reduction in fixation probability only depends on baseline density of sweeps. The scaled probability of fixation of a beneficial mutation, 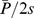, plotted as a function of the strength of selection, 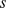. 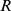 is varied along with 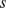, so that the ratio 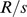 (and therefore 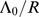) is held constant. Circles show simulation results and curves show the analytical approximation given by Eq. (8) . The scaled probability of fixation is nearly constant until 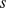 becomes large enough that unlinked sweeps become important 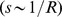. 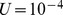, 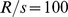 is shown in purple; 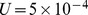, 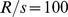 is shown in gold; 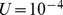, 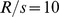 is shown in blue. 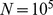 for all points and curves. Note that for 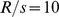, Eq. (8) slightly overestimates the amount of interference, because the chromosome is short enough that boundary effects must be considered. All simulations were run until the rate of substitution approached a steady value, and then continued until at least 1000 substitutions accumulated. The standard error is less than the radius of the points.

**Figure 4:**
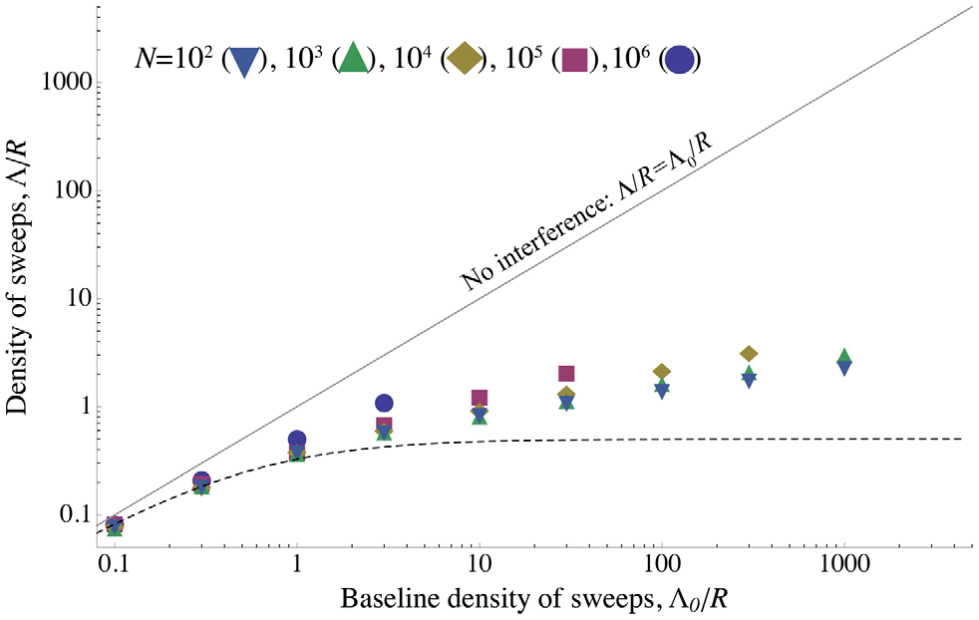
The density of sweeps as a function of the baseline density. The rate of sweeps per unit map length 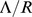, plotted against the baseline rate, 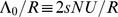. The solid line shows 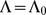, the dashed curve shows the additive approximation given by the solution to Eq. (8) , and the points show simulation results. Different kinds of points represent different values of 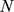; as predicted by the scaling argument, 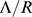 depends on 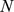 only through 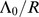. 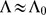 until interference becomes strong at 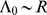, after which 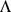 increases only slowly. While the simulated values of 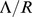 continue to increase above Eq. (8) 's “upper limit” of 0.5, they do so only very slowly, remaining 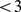 even for 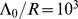. (Note that even when Eq. (8) underestimates 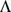, it appears that our scaling argument still holds.) Selection and map length are held constant at 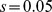 and 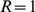 while population size 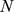 and mutation rate 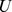 are varied. The points show simulation results averaged over 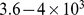 generations for 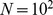 (circles), 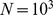 (squares), 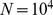 (diamonds), 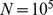 (upward-pointing triangles), and 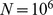 (downward-pointing triangles). For each value of 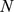, values of 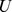 are shown up the point at which the strength of interference at which the probability of fixation falls to 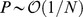 and the neutral accumulation of mutations becomes important (see Figure S7). The standard errors in the simulation results are less than the size of the points.

We still face a difficulty, however, in that the locations and times of sweeps are *not* independent: because the amount of interference varies stochastically over the genome and through time, we expect them to be overdispersed. The scaling argument will still hold if the effects of different sweeps add up (the approximation developed below), or if the distribution in scaled time and map length is non-uniform but still depends on the population parameters only through 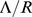. We show by simulation that the heuristic scaling argument is in fact accurate (Figure 3 and Figure 4), and that distribution of sweeps is close to uniform even for very strong interference (Figure 2). This may seem somewhat puzzling – sweeps should preferentially begin at loci and times that are experiencing less interference. However, when sweeps are rare, most of the genome experiences almost no interference in most generations, and thus little variation in the amount of interference. Conversely, when sweeps are common, most of the genome experiences substantial interference from multiple sweeps in most generations, and the stochastic variations in the amount of interference experienced from locus to locus and generation to generation are small compared to this average effect.

Above, we have shown that if interference has only a mild effect on the distribution and trajectories of common alleles that cause the most interference, then the expected scaled fixation probability depends only on the density of sweeps, i.e., that 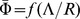 for some function 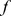. Since 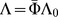, we can rewrite this as 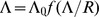, or 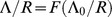, where 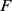 is implicitly defined by 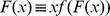; the density of sweeps 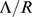 depends only on the baseline density in the absence of interference, 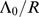.

In the above derivation, we have omitted two additional complications regarding the distribution of sweeps across the chromosome. First, for strong interference, while the rate of sweeps is nearly uniform in the middle of the chromosome, it is higher near the ends, since these end loci have fewer nearby loci to interfere with them. We will assume that the chromosome is long compared to the scale of interference, 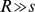 (see Figure 1 and Figure S4), so that these edge effects can be neglected at most loci. (Note that if the total map length 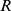 is the sum over several chromosomes, we require that each chromosome individually have a map length 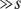.) Second, a uniform distribution over the chromosome does not exactly correspond to a uniform distribution over recombination fractions with a given locus, because the recombination fraction saturates at 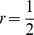. Thus, for genomes with long total map lengths, 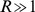, each locus will experience sweeps uniformly distributed across nearby loci, plus many more sweeps at effectively unlinked loci, which generate a variance in log fitness of 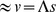. As shown in the previous section, the cumulative effect of these unlinked loci is to cause short-term fluctuations, which increase the rate of random drift by a factor 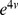 (assuming polygamy). The term in 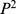 in Eq. (2) is therefore multiplied by this factor, and the fixation probability is reduced by the same factor. Combining this with the expression in the previous paragraph, we obtain an implicit equation for the rate of sweeps:
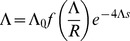
(5)Note that the density of sweeps now depends on the additional parameter 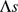, in addition to 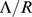; the ratio between the two parameters, 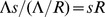, determines whether the interference experienced by a beneficial allele comes primarily from a few closely-linked sweeps (small 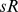) or many unlinked sweeps (large 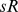).

We progressively strengthened our assumptions at each stage of the above derivation of Eq. (5) . In the end they amount to the approximation that alleles are essentially only affected by interference when rare, and cause interference only when common (although the factor 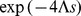 allows these assumptions to be violated for interference among unlinked loci). We can actually weaken this assumption by allowing interference to affect the trajectories of common alleles, as long as this effect only depends on 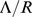. Still, for given 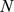, we expect that this approximation will break down for sufficiently strong interference, but that for any given strength of interference (i.e., value of 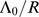), the accuracy of our scaling argument will increase with increasing 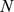, as the separation between rare and common alleles increases. The simulation results shown in Figure 3 and Figure 4 confirm that Eq. (5) is accurate over a broad region of parameter space.

### The additive approximation

We now turn to determining the function 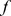 in Eq. (5) that determines the decrease in fixation probability due to interference (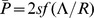
>). As mentioned above, since the number of backgrounds that must be included in Eq. (2) grows exponentially with the number of interfering sweeps, it is impractical to solve it exactly for 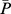. Instead, we will make the approximation that the average amount of interference experienced by a focal allele increases linearly with the density of sweeps, 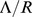; i.e., that common alleles are unaffected by interference, and that the expected effects of multiple sweeps on 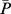 combine additively. The approximation that the effects combine additively can be justified rigorously when interfering sweeps have selective coefficients much larger than those of the sweeps being interfered with (see Text S4). Even in the case we are concerned with here, in which all sweeps have the same selective advantage 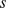, the approximation is necessarily accurate when sweeps are sufficiently rare that a new allele is unlikely to experience substantial interference from more than one sweep. In addition, we show numerically that for small numbers of interfering sweeps, their effects are roughly additive even when they occur quite close together. (See Figure S5.) Thus we will assume additive effects for the remainder of this derivation.

Under the additive approximation, the average effect of multiple sweeps on fixation probability across the genome and time is just the sum of their individual effects. The effects of a single substitution at a given genetic distance and time from a focal allele can be calculated numerically by following the coupled equations for the fixation probabilities on the two alternative backgrounds, 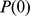 and 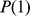 (Eq. 5 of [55]). This can then be numerically integrated over sweeps distributed uniformly over time and across the genome to find the expected fixation probability of a new mutation (Text S4):
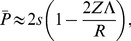
(6)where Z = 1.05. In the following, we will take 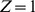 and omit it for simplicity. (A 5% difference is not worth worrying about given that our underlying model is an extreme oversimplification of a real population and that Eq. (6) is only approximately true even for our model.) Since the rate of sweeps is 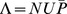 we can solve for 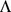:
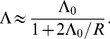
(7)(Recall that 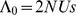.)

As explained above, we can include the effects of loosely linked loci by reducing fixation probability by a factor 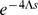, where 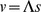 is the variance in log fitness. The result is most simply expressed in terms of this variance, relative to the baseline variance in log fitness in the absence of interference, 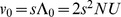, which necessarily equals the baseline rate of increase of mean log fitness. From Eqs. (1) and (6) we have:
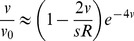
(8)As mentioned above, the product 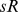 determines the importance of loosely-linked loci, relative to tightly linked loci. We now see that the condition for interference to be mainly due to the effects of tightly-linked loci is 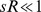. For an organism with total map length 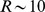, this corresponds to adaptation being primarily due to alleles with selective advantage 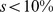. Figure 3 compares the predictions of Eq. (8) with results from individual-based simulations (see Methods) and shows that they are quite accurate up to levels of interference strong enough to reduce fixation probability by an order of magnitude. The left side of the figure shows the regime 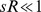 in which interference is caused by tightly-linked loci and depends only on 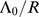; loosely-linked loci begin to interfere on the right side of the figure, where 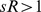.

In the limit of a very large density of incoming mutations, 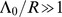, Eqs. (7) and (8) imply that 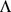 tends to an “upper limit” of 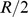. As expected from our scaling argument, this limit is independent of both population size and of the strength of selection. This upper limit implies that fixation probability should begin to scale almost inversely with 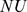 (the mutation supply) and to depend only very weakly on 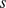 at some finite 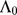 – in particular, 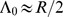. Above this limit, our approximations begin to break down and underestimate 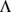, but 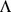 typically depends only weakly on 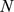, 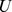, and 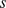 once it approaches 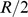. The exact form of this weak dependence remains an open question. The regime is analogous to the “multiple mutations” regime of asexual populations, and indeed results from this regime in asexual populations provide lower bounds for the rate of adaptation that increase roughly logarithmically with 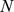, 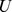, and 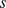 (Eq. (41) in [6] and Eq. (53) in [8], reviewed in [11]). However, these bounds are far too low to be useful for frequently recombining organisms. A better bound can be found by making the approximation that the genome is composed of many short, effectively asexual segments which interfere with each other only weakly. In this case, back-of-the-envelope calculations suggest that 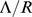 should grow at least as fast as 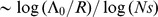, although this remains to be carefully investigated. Since beneficial mutations must be more likely to fix than neutral ones, there is an additional lower bound 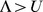 that applies when mutation is very frequent, but in this case mutations are effectively nearly neutral and may not be detectable as selective sweeps.

Figure 4 compares the above theoretical predictions with results from simulations. Parameters are chosen such that 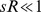, so 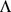 should be approximately given by Eq. (7) . As expected, for fixed 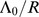, 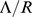 approaches the theoretical prediction as 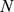 increases. Agreement is close for large populations (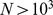) up to 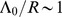, at which point the predicted rate of adaptation approaches an asymptotic limit while the simulations indicate that it continues to increase, albeit slowly. Note that the scaling argument (leading to Eq. (5) ) is more robust than our prediction for the form of the dependence on 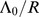 (Eq. (7)); even when the latter underestimates 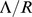, it is still true that for large 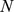, 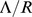 depends on 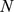 and 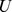 primarily through their product. For small populations and large mutation rates, the probability of fixation approaches the neutral value 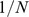, and 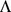 again increases linearly with 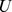 as it does for low interference, although with a much smaller constant of proportionality.

### Very strong interference: Adaptation above the limit

**Figure 5:**
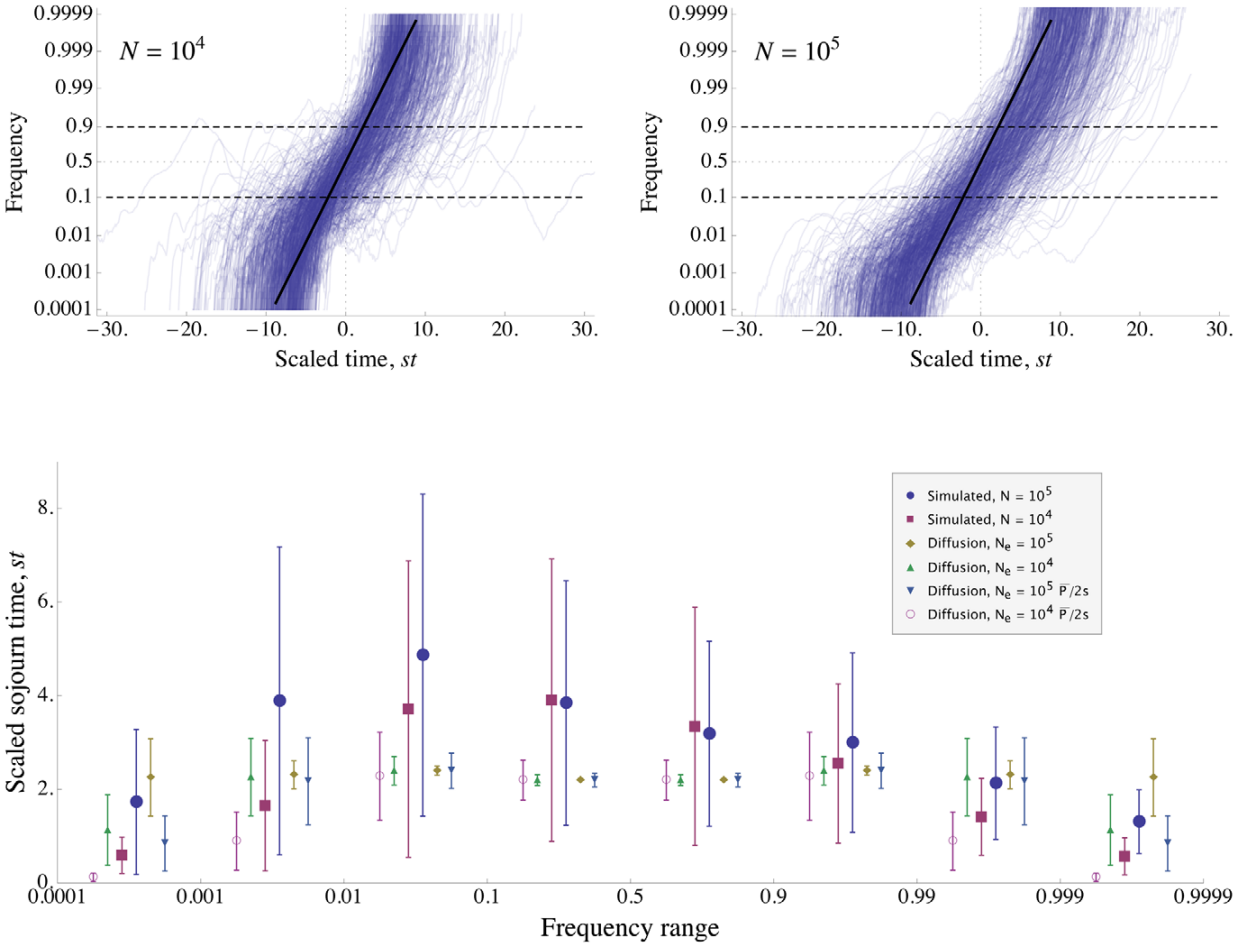
Simulation of evolution with strong interference. The figure shows data from simulated populations with mutation supply 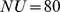. The total genetic map length is 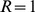 and mutations provide selective advantage 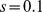. The baseline density of sweeps is 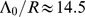, corresponding to interference strong enough that our approximation Eq. (8) for the rate of adaptation is beginning to break down. Top panels: Trajectories of 1000 example selective sweeps in a population of size 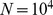 (left), and 713 sweeps in a population of size 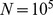 (right). Frequencies are plotted on a logit scale, so that the deterministic trajectory in the absence of interference is a straight line (shown in black). While the distributions of trajectories differ between the two populations at very low and high frequencies, they are similar in the frequency range 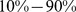 (between the dashed lines) at which sweeps cause the most interference. For each sweep, 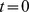 is set to be halfway between its origin and fixation, and time is scaled by 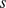. Most of the trajectories take longer to increase to high frequency than the deterministic trajectory in the absence of interference; on average, the sweeps are slowed down by interference. Most trajectories lie below frequency 1/2 at 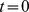, i.e., they take longer to go from frequency 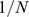 to 1/2 than from 1/2 to 1. At very low and high frequencies, the trajectories are dominated by drift and are far from the deterministic trajectory. At the intermediate frequencies at which they cause the most interference, most trajectories increase at a roughly steady rate, albeit more slowly than they would in the absence of interference. Bottom panel: Sojourn times (scaled by 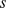) of the simulated sweeps shown in the top panels. Simulation results are compared to the distribution expected under the diffusion approximation with an effective population size of either the actual size, 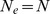, or scaled by the reduction in fixation probability, 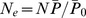. Points show mean sojourn times, while the error bars show the standard deviation of the sojourn time. (Note that this is not the standard error of the mean, which is smaller by a factor of 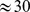.) The mean and standard deviation of the sojourn times at intermediate frequencies are approximately the same for 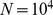 and 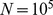. Strong interference greatly increases the variance in sojourn times. The mean increases as well, but by no more than a factor of two, much less than might be suggested by the 15-fold decrease in fixation probability. In contrast to the results in the absence of interference, the sojourn time distribution of the simulations is asymmetric about frequency 1/2. For the diffusion approximation, mean sojourn time is found from Eq. 5.53 of [50], and the standard deviation of the sojourn time is found from Eq. 27 of [105].

Since our analytical approximation Eq. (8) become inaccurate for very strong interference, we further investigated this regime by simulation. Figure 5 shows the results of a typical simulation run with parameters chosen such that there is very strong interference: 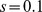, 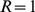, 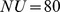. In the absence of interference, the fixation probability would be 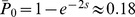, slightly lower than the weak-selection approximation of 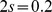, so the density of sweeps would be 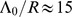. In the simulations, interference reduces the average fixation probability to 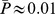, which is roughly twice as large as the fixation probability predicted from Eq. (8) . Our analytical approximations are thus beginning to break down, but the general features are still roughly correct. In particular, our basic assumption that alleles are safe from loss once they reach appreciable frequency is still true. For these parameters, loss becomes unlikely once the number of copies exceeds 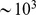, which is well below the frequencies at which the allele begins to interfere with others for 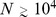. Our scaling argument assumes not only that common alleles are certain to be fixed, but also that their trajectory on the way to fixation is affected by interference in a way that depends only on the density of sweeps, 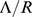. Figure 5 shows that this assumption is roughly accurate even at high interference; the distributions of sweep trajectories and sojourn times between 10% frequency and 90% frequency (the range in which sweeps cause the most interference) are similar for 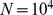, 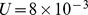 and 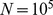, 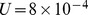.

Going beyond the scaling argument, the additive approximation used to derive Eq. (8) assumes that (i) the interference caused by different sweeps combines additively and (ii) the trajectories of alleles at intermediate frequencies are unaffected by interference. In Figure 5, we see that assumption (ii) begins to fail for very strong interference, as interference increases the sojourn time at intermediate frequencies by a factor of 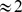 for the simulated parameters, and introduces substantial variance in trajectories. Note that this slowdown has no direct negative effect on the rate of adaptation. (If alleles spread more slowly, then each allele in a given frequency range contributes less to the rate of increase in mean fitness, but there are more alleles in every frequency range; these effects precisely cancel.) It does, however, have an indirect positive effect, because the slower rate of increase of the common alleles means that they cause less interference for new alleles than they would in isolation. If we recalculate the expected fixation probability 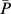 using the observed rate of increase in common sweeps (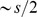) and assuming additivity of interference, we obtain the value found in the simulations. This indicates that assumption (i) is still valid even at strong interference.

Interestingly, very common alleles are less affected by interference than those at intermediate frequencies. With no interference, we expect an allele destined to fix to spend the same time increasing from 1 copy to 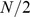 as to get from 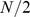 to 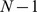 [59]. In contrast, while the sweeps in the simulation run with 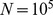 spend an average of 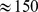 generations at frequencies less than one half, they spend only 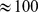 generations at frequencies greater than one half, the latter being the same as they would in the absence of interference (see Eq. 5.53 in [50]).

### Effects on neutral diversity

It is far easier to observe neutral diversity than rates of adaptive substitution: thus, it is important to know the effects of multiple selective sweeps on neutral variation. In particular, it is important to understand how the magnitude of the reduction in fixation probability of favorable alleles due to interference compares to the reduction in neutral diversity due to the “genetic draft” [60] caused by the sweeps. Since extensive molecular variation was first seen, it has been clear that in abundant organisms, diversity is much lower than expected from census numbers [61]. Maynard Smith and Haigh [62] argued that diversity may be limited in large populations by selective sweeps, an argument set out more recently by Gillespie [60],[63],[64]. Thus, we can ask whether a rate of sweeps that reduces diversity to observed levels will also cause significant interference with natural selection.

Unfortunately, it is much harder to calculate the effect of multiple sweeps on neutral diversity than it is to find the effect on fixation probability. A full description of samples of neutral genes requires that we follow their genealogy back through time, under a coalescent process that is conditioned on the changing frequencies of selected genetic backgrounds [65]. In place of an exact analysis of the full spectrum of neutral diversity, we will focus on a single quantity, the long-term pairwise rate of coalescence. Note that this single number is not enough to characterize the full effect of draft on neutral alleles, i.e., there is no one “effective population size”; see the Discussion and Figure S8.

Even calculating the pairwise rate of coalescence exactly is difficult, so we make the approximation that the rate of coalescence due to multiple sweeps is the sum of the sweeps' effects in isolation. As with our approximation that effects on selected alleles are additive, this approximation becomes inaccurate for very strong interference, when even common alleles' trajectories are affected by interference [66], [67], but must be valid when sweeps are not too common [68], [69]. In a single selective sweep with selective coefficient 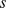, a pair of lineages at a neutral locus a distance 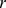 away, with 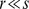, have probability 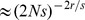 of coalescing [62], [70]–[72]. (This can be understood as the probability 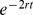 that two neutral lineages both remain associated with the sweeping allele during the time 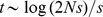 that it takes to increase from a single copy to near-fixation.) Averaging over a linear map of length 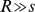, the total rate of coalescence due to nearby sweeps is 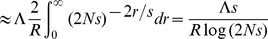 [73]. As discussed above, unlinked sweeps effectively increase the strength of drift (i.e., the rate of coalescence) by an additional factor 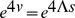, assuming polygamy [74]. Altogether, the expected time for a pair of neutral lineages to coalesce is
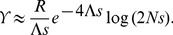
(9)Since 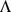 increases with 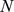, Eq. (9) implies, perhaps counterintuitively, that effective population size (as measured by heterozygosity) is a *decreasing* function of actual population size in moderately large populations, similar to the results of [63]. This can be understood by noting that when population size is large, as we assume, the rate of sampling drift is negligible, and neutral diversity must be determined primarily by selective sweeps, as Maynard Smith and Haigh originally argued [62]. Note that while increasing 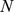 increases the number of sweeps, it also decreases the effect of each sweep on neutral diversity, because of the factor of 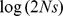 in Eq. (9) which arises from the increase in the time to sweep. Since 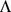 increases only slowly with 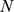 for very large 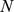, this may mean that the decrease of 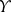 with increasing 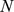 should eventually level off and perhaps even reverse.

Comparing Eq. (9) to Eq. (7) , we see that neutral diversity will be substantially reduced (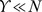) when the rate of sweeps reaches 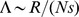, a far lower rate of sweeps than is necessary to interfere with adaptive alleles (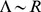) for 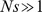. (Sweeps at unlinked loci affect neutral and adaptive alleles similarly, but closely-linked loci are generally likely to be the main cause of draft; see Discussion below.) Thus, at low densities of sweeps, neutral diversity is much more affected by sweeps than is fixation probability. In contrast [73], argued that the opposite should be true, since the characteristic genetic map distance over which a sweep reduces neutral diversity (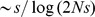) is smaller than the scale over which it causes interference (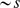). However, this difference in length scales is not very big −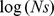 is unlikely to approach 100 in natural populations – and thus has only a mild effect. Our results indicate that some populations (experiencing weak interference) may be able to adapt much more rapidly than would be expected from measurements of “
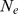
” based on heterozygosity. (This may be the case for Drosophila – see below and [75].) On the other hand, even populations experiencing strong interference may maintain substantial neutral diversity. This is because the loss of diversity depends on the actual density of sweeps 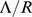, which plateaus when interference becomes strong, rather than on the baseline density 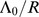.

**Figure 6:**
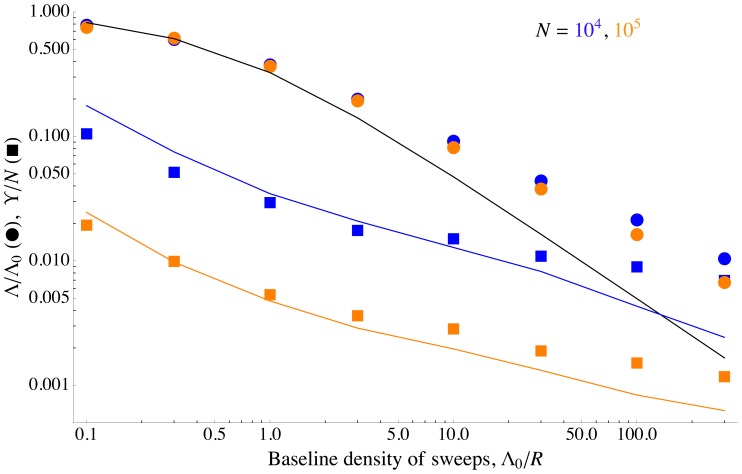
Differing effects of sweeps on selected and neutral alleles. The scaled fixation probability of beneficial alleles and scaled neutral diversity as a function of the baseline density of sweeps 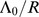. Points show simulation results, curves show analytical approximations. The circles and the black curve are the scaled fixation probability 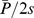, and show the same data as in Figure 4. The squares and colored curves show the scaled neutral diversity, 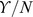. At small 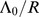, beneficial alleles do not interfere with each other, but still reduce neutral diversity substantially. However, increasing 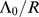 to larger values has little additional effect on neutral diversity, both because interference limits the increase in the number of sweeps (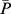 decreases), and because the combined effect of overlapping sweeps on neutral diversity is less than the sum of their individual effects (the squares lie above the additive analytical approximation). The analytical approximations match the simulation results up to strong interference (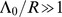), at which point they begin to break down. The squares are the averages over 100 simulation runs; see the Methods for how 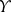 was measured. The colored curves show Eq. (9) for 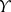 as a function of 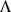, with 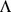 taken empirically from the simulations. The mutation rate 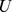 is varied, with other parameters held constant at 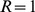, 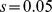, and 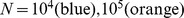. For these parameter values, essentially all interference is caused by tightly-linked loci.

As shown in Figure 6, Eq. (9) is roughly in agreement with the rate of coalescence observed at a neutral marker locus in simulated populations. Figure 6 also shows the simulation results and the analytical approximation ( Eq. (8) ) for the rate of adaptation, in terms of reduction in the probability of fixation, 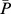. We can clearly see the different scalings discussed above: while both neutral diversity and 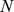 decrease as the baseline density of sweeps 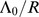 increases, they do so in opposite ways. Beneficial mutations are nearly unaffected by interference until 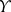 approaches one, at which point 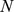 drops rapidly. Neutral diversity, on the other hand, is strongly reduced even at small 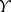, but is nearly independent of 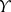 for 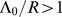, precisely because interference limits the increase in 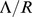 in this regime. In addition, for very high rates of sweeps, interference between successful sweeps causes their effect on coalescence to be sub-additive, further preserving neutral diversity [66], [67]; a similar effect also limits the reduction in neutral diversity caused by background selection [76], [77].

### Distribution of selective advantages

**Figure 7:**
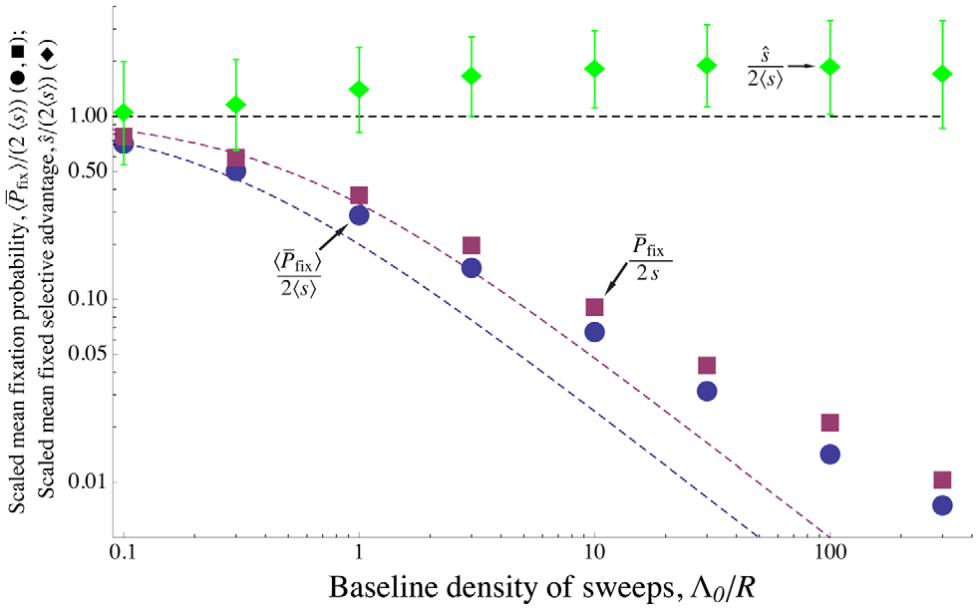
Effect of interference among alleles with a distribution of selective advantages. Simulation results for scaled mean probability of fixation 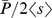 for mutations with exponentially distributed selective advantages (blue circles) and scaled mean selective advantage for successful mutations 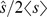 (green diamonds), as a function of the baseline density of sweeps 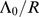 – i.e., the amount of interference. The purple squares shows 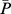 for the same parameter values, but with all mutations conferring an identical selective advantage 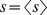. Allowing for a distribution of selective effects makes little difference in the rate of sweeps, 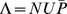, and the mean selective advantage of sweeps stays close to 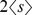 (dashed black line), even for strong interference. The theoretical predictions Eqs. (7) and (13) (purple and blue dashed curves, respectively) are accurate for weak interference, but underestimate fixation probability with strong interference. The mutation rate 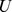 is varied, with other parameters held constant at 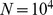, 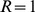, and mean selective advantage provided by a mutation 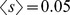. All points are averages over 5000 simulated generations. Error bars on the top curve show the standard deviation of 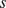 for successful mutations. The standard errors are less than the size of the points.

Above, we have focused on the case in which all beneficial mutations provide the same selective advantage 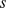. Using simulations, we have also investigated the effect of allowing exponentially distributed selective advantages. ([10] and [78] conduct similar studies for asexual populations.) Figure 7 shows that for both weak and strong interference, allowing for variation in 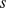 makes little difference to the rate of adaptation. Populations with an exponential distribution of mutational effects with mean 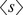 evolve only slightly slower than populations with a fixed value 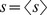, and show nearly the same scaling with the strength of selection.

**Figure 8:**
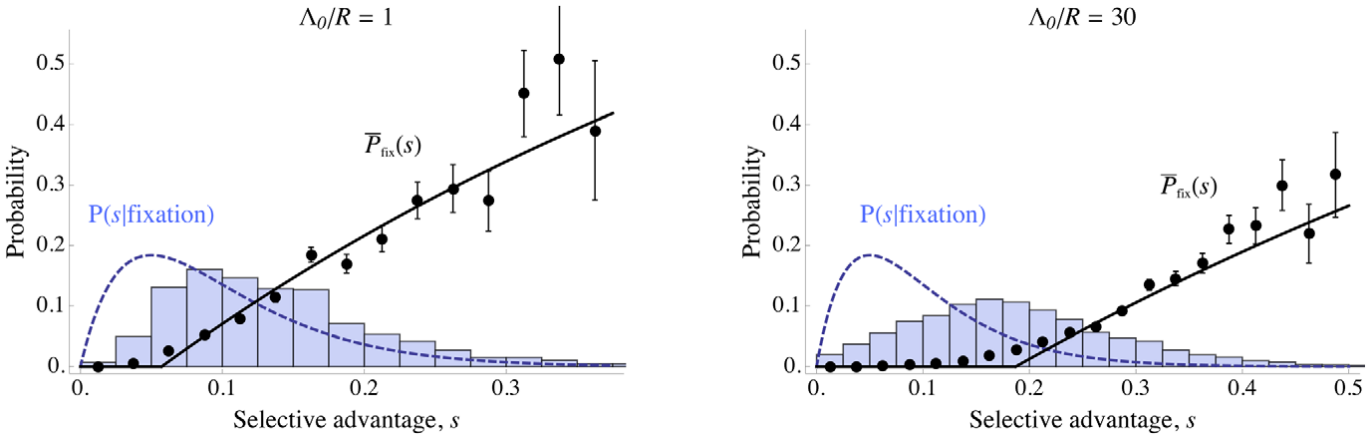
Effect of interference on distribution of successful mutations. Solid curves and points show the probability of fixation of a mutation as a function of its selective coefficient, 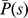. Histograms and dashed curves show the distribution of selective coefficients of fixed mutations. The left panel shows results for moderate interference (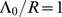), while the right panel shows high interference (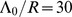). Mutations with small effects are strongly affected by interference, while large-effect mutations are nearly unaffected; this biases the distribution of successful mutations towards larger effects. The distribution of mutational effects, 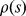, is exponential with mean 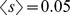. Solid curves show the analytical approximation Eq. (12), corrected for the effect of unlinked loci and the saturation of fixation probability as 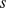 approaches 1 (see Text S4). Dashed curves show the predicted distribution of selective coefficients of fixed mutations in the absence of interference, 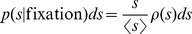, with 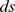 set to the width of the histogram bins. Parameters are 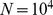, 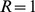, and 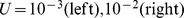. Points and histograms are averages over 5000 simulated generations; error bars show the standard error. Only a few mutations in the simulated populations had very high values of 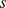, so the estimated probabilities of fixation for these high values are noisy. Note that the horizontal scales of the left and right panels are different.

Figure 8 shows that alleles with small selective advantages are much more affected by interference than those with large selective advantages. To understand this, consider the probability of fixation of an allele with advantage 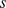, 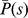, given the distribution 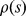 of mutational effects. (For the exponential distribution we consider here, 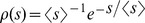.) If the effects of multiple interfering sweeps are additive, then following the argument given in Text S4 , we can write the probability of fixation as
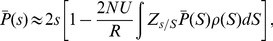
(10)where the factor 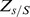 depends only on the ratio of the selective coefficients. Eq. (10) approaches 0 at some 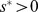; alleles with selection coefficients 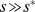 are nearly unaffected by interference, while those with lower 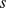 are strongly affected. (Obviously, the Eq. (10) only applies to values of 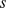 above this cutoff 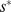; we discuss weakly-selected alleles below.) 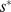 can be understood as the rate at which the focal allele is knocked back by interfering sweeps [79].

In Text S4, we find that the interference coefficient 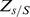 is approximately
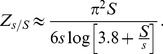
(11)(See Figure S6). While Eq. (11) can be used to solve Eq. (10) numerically, to find an analytical approximation we will instead make the crude approximation that 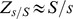. This is accurate for 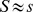, but overestimates interference for 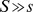. With this approximation, the probability of fixation is 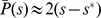, with cutoff selective coefficient 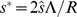, where 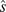 is the mean selective advantage of alleles that successfully sweep. Approximating 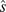 by its baseline value, 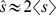, we have
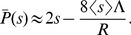
(12)
Figure 8 shows that Eq. (12) is accurate for strongly-selected alleles. While we do not currently have a simple analytic expression for the fixation probability of alleles with moderate selective advantages 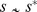, the equivalent expression for asexual populations has recently been found by [80], and it may be possible to extend this analysis to sexual populations.

Solving Eq. (12) for the overall rate of sweeps gives
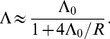
(13)Comparing Eq. (13) to Eq. (7) , we see that the amount of interference is twice that of a population with a fixed selective effect 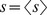. Figure 7 shows that Eq. (13) is accurate for weak interference, but is even less accurate than Eq. (7) for strong interference.

### Weakly-selected alleles

To find the probability of fixation of weakly-selected alleles that primarily experience interference from alleles with much larger selective coefficients, we can take the small 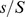 limit of Eq. (11) , 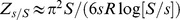 [79]. Assuming that the selective coefficients of mutations that succeed in fixing are clustered fairly tightly around their mean value, 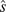 (as they are in the simulations shown in Figure 7 and Figure 8), the fixation probability of the weakly-selected alleles is approximately
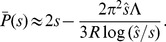
(14)


Eq. (14) predicts that there is another, lower, selective coefficient 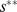 below which alleles are nearly neutral. Eq. (14) breaks down as 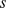 approaches 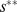; our derivation assumed that an allele's fate is decided when it is rare, which applies only when selection is strong relative to drift (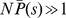). More weakly selected alleles must drift nearly to fixation before selection becomes effective and they are safe from extinction. Since their fate is decided over time scales similar to that of neutral alleles and by similar dynamics, we expect them to be affected similarly by interference. Thus, the degree of adaptation will depend on 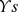, where 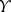 is given by Eq. (9) . ( Eq. (9) still approximately holds for an exponential distribution of sweep strengths under weak interference, with 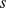 replaced by 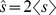.) For this heuristic argument to agree with Eq. (14) for 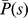, we must have 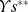
<∼1; comparing Eqs. (9) and (14) , we see that this condition is satisfied. However, this is far from conclusive, and the dynamics of weakly-selected alleles should be investigated further. Neher and Shraiman [30] conduct a more detailed analysis for the infinitesimal model, and obtain qualitatively similar results.

## Discussion

### Summary of results

When many beneficial alleles are sweeping through a population, interference among them may greatly retard adaptation. In this case, the rate of adaptation may be primarily limited by the rate at which recombination can bring beneficial alleles together in the same genome. A scaling argument shows that for a given distribution of selection coefficients, the density of successful substitutions per generation per chromosome arm, 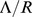, is a function *solely* of the density that would be expected in the absence of interference, 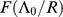, and does not depend on the beneficial mutation rate 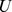, the total genetic map length 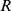, the population size 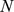, or strength of selection 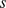 separately. When mutations have equal effects, we obtain an explicit approximate formula for the density of substitutions, 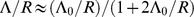. This implies that there is an “upper bound” to the density of sweeps, 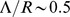. When the population variance in log fitness, 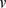, is large, interference from unlinked loci further reduces the rate of sweeps by a factor 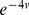 or 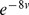, depending on the mating system. However, for 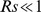, most interference occurs between linked loci separated by a map distance 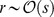.

Simulations show that the scaling argument is accurate over a broad range of parameters. Numerical calculations and simulations show that the explicit formula for 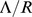 is accurate for up to a few interacting sweeps, but substantially underestimates the rate of adaptation when there are many closely-linked, concurrent sweeps. The simulations indicate that the rate of adaptation continues to increase above the “upper bound” as 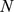 and 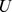 increase, perhaps logarithmically; however, this increase becomes so slow that 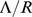 is unlikely to greatly exceed one in most populations. Simulations also indicate that the assumption that all mutations have the same effect can be relaxed without affecting the key results. Genetic draft greatly reduces neutral diversity when the density of sweeps exceeds 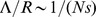, far lower than the density needed to cause interference; however, even when sweeps are dense enough to cause extreme interference, neutral diversity is not reduced by much more.

### Relation with previous work

Several authors have recently studied interference among unlinked loci [23], [24], [26], [27] . Cohen et al. [23], [24] and Rouzine et al. [26] consider models in which the total number of possible adaptive substitutions is fixed, so that sufficiently large populations reach a maximum rate of adaptation, a different situation from the one we consider. However, [26] do show that the infinitesimal model used here is a good approximation to the dynamics of unlinked loci for a broad range of parameters. Neher et al.'s model [27] includes mutations and is more similar to ours. However, [26], [27] consider only facultative sexuals and assume a small rate of outcrossing, 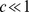. As mentioned above, our infinitesimal model can be straightforwardly extended to a similar case, in which individuals outcross only every 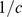 generations, by scaling selective coefficients by 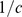, i.e., by replacing 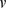 by 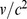. This implies that the boundary between weak and strong interference is at 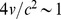, consistent with [27]. [27]'s result for the weak interference regime (the second line of their Eq. 12) is the same as predicted by our Eq. (1) . For strong interference, our scaled Eq. (1) has the limit 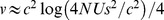, somewhat different from the first line of their Eq. 12 (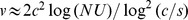 in our notation). Both predict only a logarithmic increase in 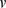, but the dependence on the underlying parameters is different. This is because in their model rare, extremely fit genotypes can produce large clonal lineages without being broken up by recombination, whereas in ours all lineages eventually recombine. Their model is more appropriate for organisms that have a small chance of outcrossing in every generation (which is most likely for bacteria and viruses, and also some eukaryotes), while ours applies to organisms that outcross at regular intervals between rounds of asexual reproduction (as is the case with some eukaryotes).

Both [27] and [26] ignore the possibility of varying degrees of linkage among loci (i.e., there is no genetic map). This is a natural model for bacteria in which recombination typically involves the replacement of short stretches of DNA, and most loci therefore have the same recombination fraction with each other. However, in viruses and eukaryotes, recombination is primarily due to crossovers, as in our model. In this case, adjusting our Eq. (8) for facultative sexuals outcrossing at frequency 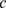 gives
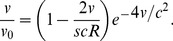
(15)Eq. (15) indicates that linked loci are the primary source of interference when 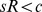, which we expect to be true for many populations. Thus, we expect interference among beneficial mutations to be more prevalent than predicted by previous studies. Considering both the differences between the models of facultative sex discussed in the previous paragraph, and the differences between the models of recombination, the models of [26], [27] are generally more appropriate for bacteria, while ours is generally more appropriate for eukaryotes with an obligate outcrossing stage in their life cycle. For viruses and eukaryotes that outcross rarely and randomly, their models do a better job of capturing interference among unlinked loci, and are therefore more appropriate for organisms with 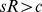, while ours is better when most interference is from tightly-linked loci (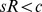).

Neher and Shraiman [30] have recently extended [27] to consider the effect of genetic draft on neutral diversity. Although they consider different measures of diversity than we do, their results are qualitatively similar to those of our infinitesimal model ( Eq. (9) for 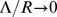, and scaled by the outcrossing frequency): draft is significant when the variance in log fitness exceeds the square of the outcrossing rate, 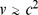, i.e., 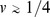 for our model of obligate sexuals. A similar result was also derived by Santiago and Caballero [74]. Note that this is the same threshold value at which interference from unlinked loci begins to affect advantageous alleles. In our model of a linear genetic map, in contrast, the rate of sweeps necessary to create significant draft is much lower than the rate needed to cause strong interference: Eq. (9) predicts that that 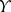 will be much less than 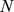 for 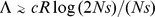, typically a much weaker condition than 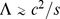. If we consider the case of HIV within-host evolution addressed by [30], taking the frequency of outcrossing to be 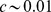, the map length to be 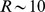, and typical positive selective coefficients to be 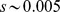 [29], [81], [82], we see that for any reasonable population size (roughly, 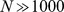), the threshold value of 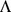 at which draft from linked sweeps becomes important is smaller than that at which draft and interference from unlinked sweeps become important. Santiago and Caballero [83] extend [74] to allow for the effect of a genetic map; their framework can be used to derive the roughly the same threshold rate of sweeps 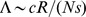, but drastically underestimates 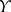 for the draft-dominated populations described by Eq. (9).

### Deleterious mutations

Because deleterious mutations are far more frequent than beneficial mutations, it is important to consider how they affect our results. The effect of unlinked deleterious mutations is easy to incorporate into the infinitesimal model by repeating the analysis using the exact expression for the rate of increase in mean log fitness, including the direct effect of new mutations, 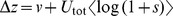, where in the second term 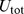 and the expectation over 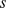 include deleterious mutations. Unlinked mutations simply increase the effective strength of drift and can be described as reducing the effective population size. The effect of linked deleterious mutations can also easily be included when deleterious mutations and sweeps are not so common that they substantially reduce the efficacy of negative selection. In this case, deleterious mutations with selective disadvantage 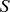 occurring at a genomic mutation rate 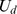 reduce fixation probability at linked sites by a factor 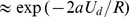, where 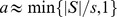 [55]. In contrast to the effect of unlinked loci, this clearly cannot be captured by a reduction in a single effective population size, as beneficial alleles of different effects experience different amounts of interference; since 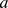 decreases with 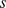, strongly selected alleles experience less interference from background selection, just as they experience less interference from other sweeps (Figure 8). Background selection has the largest effect when there are many linked deleterious alleles, but in this case the deleterious alleles interfere with each other and the situation becomes more complicated [76]. This case and the one in which deleterious alleles experience strong interference from sweeps remain to be investigated analytically.

### Population subdivision

It is important to consider how population subdivision interacts with interference in determining the rate of adaptation. When few favorable alleles enter in each generation, so that 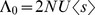 is small, the rate of adaptation increases in proportion to population size, 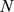, while Hill-Robertson interference leads to diminishing returns for increasing population size. This appears to suggest that a subdivided population, consisting of many small demes, might adapt more efficiently. However, note that for an allele to fix in the entire population, it must fix in every deme; in addition, other alleles may fix only locally before going extinct. Thus, every deme experiences at least the same rate of sweeps, 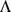, as would a single panmictic population. Thus, strong population subdivision will increase interference among sweeps, most of which enter the local deme by migration, rather than by mutation. [56], [57] showed that with conservative migration, and in which each deme contributes according to its size, the fixation probability of a favorable allele is unaffected by population structure. We believe that this result does not carry over to the effects of multiple sweeps, and that overall, the fixation probability will be reduced by subdivision. This has been found to be true for asexual populations [84], but remains an open question in sexual populations.

### Likely strength of Hill-Robertson interference

It is unclear how important the Hill-Robertson effect due to selective sweeps is in biological populations, both because it is difficult to measure the local rate of adaptive substitutions and because the expected amount of interference had not been determined theoretically. Above, we addressed the second question, and found that interference between substitutions becomes important as the rate of adaptive substitutions approaches one per Morgan every two generations. Here we briefly discuss what is known about the first question, and what this implies for the relevance of Hill-Robertson interference from sweeps.

#### Artificial selection

Does Hill-Robertson interference limit the response to strong artificial selection on sexual populations? At first, the response must be due to standing variation, and may depend on alleles initially in many copies. (However, many microbial evolution experiments start with very little standing variation; this situation is discussed in Text S5.) The reduction in fixation probability considered here is hardly relevant in this initial phase, though negative linkage disequilibria between favorable alleles will slow down the response. However, even completely homogeneous populations respond to selection after an initial delay, showing that there is a high rate of increase in genetic variance due to new mutations, 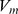: typically, 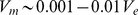, where 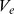 is the non-genetic component of the variance in the trait [53]. Thus, after some tens of generations, new mutations will start to contribute, and ultimately, the rate of fixation of such mutations limits the selection response [85], [86]. In the absence of Hill-Robertson interference, this could in principle lead to an extremely high rate of adaptive substitution. An allele with effect 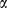 on a trait with total phenotypic variance 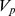 has selective advantage 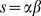, where 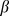 is the selection gradient, which is typically of order 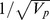. (For example, if the top 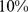 are selected, 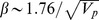). Therefore, the baseline rate of substitution due to mutations of effect 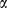, arising at net rate 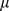 per genome per generation, is 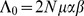. Since 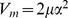 (assuming that mutations are equally likely to increase or decrease the trait under selection), this can be rewritten as 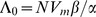. Selection can pick up alleles with effect larger than 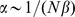, and so substitutions could occur at up to 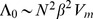. Using the middle of the estimated range of 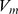 from [53] and assuming 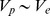 gives 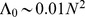. Thus, even moderately-sized populations could in principle sustain extremely high baseline rates of adaptive substitution, both because they generate large numbers of mutations, and because selection can be effective on alleles of small effect. It seems that populations under artificial selection could easily be in the regime 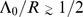 in which Hill-Robertson interference is strong.

It is difficult to determine if Hill-Robertson interference has limited the response in past artificial selection experiments, largely because we still have very limited understanding of the causes of mutational heritability, and of the genetic basis of selection response [87], [88]. Sequencing of genomes from pedigrees and from mutation accumulation lines has given good estimates of the total genomic mutation rate [89], but we do not know what fraction of these mutations have significant effects on traits, or the distribution of these effects. In a classic experiment, selection for increased oil content in maize has caused a large and continuing response; after 70 generations, Laurie et al. [90] identified 50 QTL responsible for 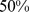 of the genetic variance in a cross between selected and control lines, implying 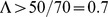 on a map of 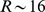. The effective population size here is extremely small (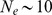) and so much of this response must be due to new mutations [91], so the density of sweeps is 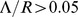. Thus, it is unclear if Hill-Robertson interference has been important, but it would likely at least be an obstacle to attempts to increase selection response further via increasing 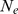. Burke et al. [92] have recently identified many regions (“several dozens”) that show consistent changes in allele frequencies across replicate populations of *Drosophila melanogaster*, selected over 600 generations for accelerated development. However, these do not show the complete loss of variation expected for a classic sweep, even though most of the response over this long timespan should be due to new mutations. This may be because the causal alleles have very small effect, and have not yet fixed – implying that the long-term rate of adaptive substitution could be very high. (Similarly, there are hardly any fixed differences between human populations on different continents, despite extensive adaptive divergence [93].) Whole-genome sequencing of selection experiments may soon give us a much better understanding of the rate at which adaptive mutations are picked up by selection. At present, however, selection experiments are inherently limited to detecting at most fifty or so sweeps over some tens of generations, and so without longer-running experiments we will not know how high the long-term rate of substitution may be.

#### Natural populations

To see whether Hill-Robertson interference could plausibly limit adaptation or diversity in natural populations, consider the evolution of Drosophila since the divergence between *simulans* and *melanogaster*. Taking the rate of adaptive substitutions (including those in non-coding regions) to be 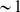 every two years [94] and the generation time to be roughly two weeks (Table 6.11 in [95]), we find that the per-generation rate is 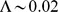. The total sex-averaged map length is 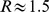 [96], so the density of substitutions is 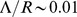, well below the interference threshold. Observed levels of neutral diversity [97], [98] and per-base mutation rates [99] suggest that the (long-term) effective population sizes of *Drosophila melanogaster* and *simulans* are roughly 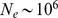. Taking the above estimate of 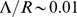, and considering the effect of the 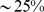 of the sweeps that Sattath et al. [100] estimate to have selective coefficients 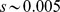, Eq. (9) tells us that this corresponds to an actual population size of about 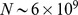, consistent with the estimate of [75]. This suggests that Drosophila may lie in the intermediate region illustrated in Figure 6, in which sweeps are frequent enough to suppress neutral diversity, but not frequent enough to interfere with each other. However, the estimates of the underlying parameters are very uncertain; see Sella et al.'s review [101].

The above back-of-the-envelope calculation probably understates the importance of the Hill-Robertson effect in evolution for several reasons. First, our results indicate that for many populations interference occurs primarily between tightly linked sites, so that it is the local, rather than genome-wide, density of sweeps that is constrained; thus, if positively selected loci are unevenly distributed across the genome, the genomic density of substitutions will underestimate the amount of interference. Similarly, regions of the genome with low recombination rates may experience increased interference. Second, we find that the interference is mainly caused by selection driving alleles from moderately low frequencies to intermediate frequencies, with relatively little interference caused by very rare alleles reaching low frequencies or common alleles going to fixation. This means that soft sweeps, partial sweeps, and polymorphic loci undergoing fluctuating selection could contribute substantially to the Hill-Robertson effect without showing up as fixed differences between species. Third, local populations may experience a substantially higher rate of selective sweeps than indicated by the species-wide molecular clock. Most importantly, organisms that have a linear genome but do not outcross every generation, such as selfers and many viruses, are more likely candidates for experiencing Hill-Robertson interference among selected alleles than are obligate out-crossers like Drosophila. For instance [29], find that interference likely reduces the rate of adaptation of HIV in the chronic stage of infection by a factor of roughly 4.

### No single effective population size

The effect of selection on surrounding genetic variation is often described as a reduction in an “effective population size.” Our results show that lumping drift and interference together in a single number in this way is generally misleading. Drift and unlinked variance in fitness dominate short-term stochasticity in allele trajectories, while the effect of linked sweeps becomes important over longer time scales (see Figure S8). This means that the “effective population size” estimated from the common, old alleles that dominate heterozygosity is likely to be very different from the relevant quantity for rare, young alleles. Thus, estimates of the strength of selection against rare alleles in, e.g., Drosophila may be systematically off by orders of magnitude. This contrast between drift dominating at short time scales and draft dominating at longer ones may also be used to estimate the amount of interference in natural populations from site frequency spectra [30], [102].

### Hill-Robertson interference and the evolution of recombination

If adaptation is limited by the rate of recombination, then there should be strong selection to increase it. Barton [46] outlined the results derived here, and their implications for the debate over the maintenance of sex and recombination. Our results imply that if recombination does limit adaptation, then increasing recombination would increase fitness in proportion. However, a modifier of recombination would itself gain an advantage only to the extent that it remained associated with the favorable combinations of alleles that it helped generate. With loosely linked loci, its advantage would be of the same order as the fitness gain across one generation; on a linear map, a recombination modifier would gain only from tightly linked alleles, less than 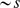 map units away; the net effect would seem likely to be very small [19]. Yet, recombination does increase significantly in artificially selected populations [103], and simulations of populations adapting at many loci show that selection for increased recombination can be strong [28], [104]. In addition, deleterious mutations are also likely to create Hill-Robertson interference, increasing selection for recombination [18], [76]. An analytical description of the evolution of modifiers of recombination rates in populations experiencing substantial genome-wide interference remains to be found.

## Methods

### Simulations

Simulations of multilocus evolution are computationally demanding, because we must follow very many individuals, and very many alleles. Because many alleles segregate simultaneously, there are typically a very large number of possible genotypes. Therefore, we must follow individuals rather than genotype frequencies, which limits the size of population that can be simulated.

The model described above was simulated using the C programming language. To minimize memory use, only a single copy of each mutation is stored; each individual is an array of references to mutation objects. Each mutation object records its location in the genome, its effect on fitness, and how many organisms in the population carry it; once this count drops to zero or rises to 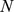 copies, the mutation object is removed from each individual's record, and is noted as fixed or lost. This memory management scheme allows simulations of more than 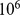 individuals to be run on a modern desktop computer. Individual fitness was calculated as the product of contributions 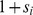 from each mutation; in most simulations, 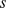 was constant. In each generation, 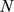 pairs of parents were chosen independently, with probability proportional to their fitnesses, with each pair producing a single offspring individual. (This is the “polygamous” model described above.) The offspring genome was generated using a Poisson number of uniformly distributed crossovers, with expectation 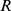, and a Poisson number of new mutations occur in each generation, with expectation 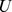. The Mersenne Twister algorithm (MT19937) was used to generate random numbers.

All simulations began with purely wild-type populations which then accumulated mutations. All data used in figures are from after the rate of substitution approached a steady value, which took 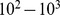 generations, depending on the parameters.

### Neutral diversity

To determine the neutral diversity, we adjusted the model described above using a method similar to [66]. After 1000 generations of evolution to allow the populations to approach a steady rate of adaptation, we “painted” each individual with a unique neutral marker allele at a locus in the middle of the chromosome, and then continued the simulation until one marker allele fixed. We then calculated the heterozygosity at the marker locus in each generation, defined as 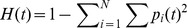, where the 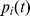 are the frequencies of each of the marker alleles in generation 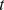. From this we estimated the rate of coalescence using the mean long-term rate of decrease in heterozygosity, 
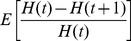
, averaged over 100 simulations run until all diversity at the marker locus was lost (see Figure S8).

### Numerical calculations

Numerical analysis was performed using *Mathematica*. The code will is available in Protocol S1.

## Supporting Information

Figure S1Reduction in the rate of adaptation caused by uncorrelated fitness fluctuations. The rate of selective sweeps 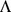 when fitness fluctuations are uncorrelated across generations, as a function of the baseline rate in the absence of fitness fluctuations, 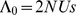. The dots show simulation results, the solid curve shows the theoretical prediction 
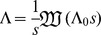
, and the dashed line shows 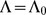. The selective advantage of mutant alleles is 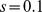. For the simulations, population size is held constant at 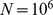 while mutation rate 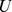 is varied. The points are the average rate of sweeps over 1000 simulated generations, discarding the first 200 generations.(TIF)

Figure S2Interference among unlinked loci. The reduction in fixation probability due to inherited variation in fitness, under the infinitesimal model. The scaled fixation probability 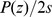, of an allele with advantage 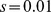 that arises in a haploid individual with value 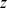 is plotted against 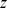 on a log scale. The lines show the predictions 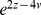 for polygamy (left panel) and 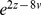 for monogamy (right panel); the variance in log fitness is 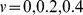 (left) and 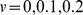 (right), running from top to bottom. Points show estimates from simulations of the infinitesimal model; these were run until at least 400 lineages reached a size greater than 5000 individuals, at which point they were considered fixed. Standard errors are less than the size of the points.(TIF)

Figure S3Interference caused by a single sweep over time. The scaled loss of fixation probability, 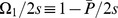, of a new allele with advantage 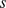 caused by the sweep of an allele also with advantage 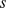 at another locus, as a function of the scaled time 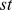 between the midpoint of the sweep and the birth of the focal allele. (Negative times correspond to the focal allele arising before the interfering sweep reaches frequency 1/2.) The curves show the effect of interfering loci at scaled genetic distance 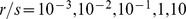 (moving down). Note that for all values of 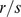 the amount of interference peaks at 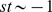, and falls off as 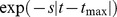 away from this maximum. Note also that for 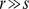, interference peaks at less than 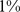 reduction in fixation probability, while for 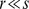, interference depends only weakly on 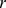. 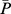 is calculated numerically from Eqs. (2) and (3) .(TIF)

Figure S4Total interference caused by a single sweep at different genetic distances. The dotted line shows the total interference caused by a selective sweep at a locus a map length 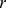 away. Both the sweep and the alleles with which it is interfering have selective adavantage 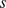; the interference 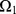 then depends only on 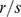. The points are obtained by numerically solving and integrating Eqs. (2) and (3) . The solid blue line shows 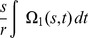; we see that the dotted line falls off faster than 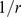 for 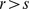, while falling off slower than 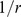 for 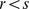, indicating that the total interference integrated over loci (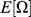, see 4 ) is dominated by 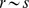. For 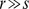, the slope approaches 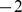 on this log-log plot (purple line), as predicted by Robertson [54] and by our argument for unlinked loci above.(TIF)

Figure S5Reduction in fixation probability due to a pair of sweeps. Numerical results for the reduction in fixation probability caused by two sweeps, as a function of the distance between them. Both plots show dimensionless scaled variables, so that they are independent of the strength of selection 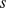 in large populations (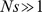). Solid curves show results for a “finite population”, in which the sweeps begin in complete negative linkage disequilibrium at frequency 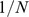, and then follow deterministic trajectories. Dashed curves show the results for an infinite population in which the sweeps are in linkage equilibrium. The dotted curves shows the summed effect of two sweeps that occur very far apart in time, so that there is no interaction. At all map distances, the amount of interference is close to that of two independent sweeps, even allowing for linkage disequilibrium. The curves are obtained by numerically solving and integrating Eqs. (2) and (3). Left panel: The net reduction in fixation probability at a single locus caused by two sweeps, 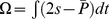, is plotted against the scaled map distance 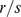 between the sweeps and the focal locus, which lies midway between them. 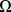 is averaged over possible time intervals between the sweeps ranging from 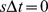 to 5; 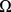 depends only weakly on this time interval, varying by less 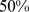 betweeen 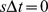 and 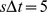 for each of the map distances. The solid curve is for population size 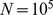. Right panel: The scaled net reduction in fixation probability over the whole genome caused by a pair of simultaneous sweeps, 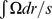, where the integral is over the map position of the new mutation. This is plotted against the scaled map distance between the two sweeps. The solid curve is for population size 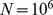. The effects of linkage disequilibrium and interaction between the sweeps are always small, but they are largest for 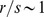, when the region of the genome experiencing substantial interference from both sweeps is maximized. (At larger values of 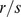, the sweeps become approximately independent.)(TIF)

Figure S6Interference coefficient 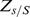. 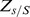, defined in Eq. (10) , describes how much sweeps with selective coefficient 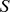 interfere with alleles with selective coefficient 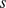. Points show the result of numerical integration of Eq. (6) of [55]. The blue curve shows the 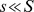 approximation from 4 . The purple line shows the 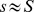 approximation 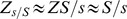. These two approximations are valid for 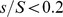 and 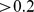, respectively. The black curve shows the combined approximation, Eq. (11) . The numerical results are expected to be overestimate 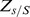 (i.e., the amount of interference) for 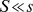, but even so predict that the interference will typically be negligible.(TIF)

Figure S7The density of sweeps as a function of the baseline density. A more detailed version of Figure 4, including the accumulation of mutations by neutral drift (combined theoretical predictions shown by dashed curves). For small populations (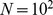 for the parameters shown), drift overwhelms selection once interference becomes strong, and “adaptive” mutations become effectively neutral. In this regime, 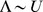, and our scaling argument breaks down. In larger populations (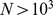), the probability of fixation remains much higher than 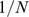 even for strong interference. This parameter regime remains to be described analytically, but it appears that the scaling argument is still a good approximation.(TIF)

Figure S8Decrease in neutral diversity over time. Decay of heterozygosity, 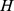, over time at a neutral locus, for a population in which every individual starts with a unique marker and there is no further mutation at the marker locus. The right panel shows the same data as the left, but on a log-logit scale. Initially, heterozygosity decays by neutral drift, decreasing at a rate of 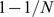 per generation, but then decays faster due to genetic draft. Since the stochasticity introduced by genetic draft has different strengths over different time scales, it cannot be fully described by adjusting a single “effective population size.” Black dots are averages over 100 simulation runs, with error bars showing the standard error. The blue curves show the heterozygosity expected for a population evolving neutrally in continuous time, 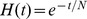. The red curves are a fit to the simulation data for 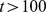, when the heterozygosity has approached its long-term rate of decrease: 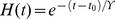, where 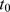 is an offset to account for the initial slow decrease in 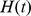. The inferred value 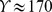 is insensitive to the exact fitting method used. Parameters are as in Figure 6, with 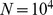 and beneficial mutation rate 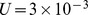, corresponding to 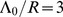. (The curves for other values of 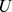 are qualitatively the same.)(TIF)

Figure S9Variation in rate of increase of mean fitness. The increase in mean log fitness per generation, 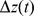 (left panel), and the auto-correlation function 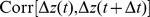 (right panel) for a simulated population. 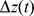 is negatively auto-correlated on the time scale 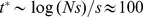 over which alleles go from a few copies to the frequency 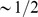 at which they cause the most interference. The population was initially monomorphic, and thus 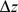 starts low, then spikes as the first wave of mutations reach intermediate frequencies. This wave then strongly interferes with new mutations, causing a later decrease in 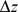; etc. The population parameters are as in Figure 5, with 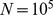. Data in the left panel are averaged over a 5-generation window. Excluding the first 500 generations leaves the auto-correlation shown in the right panel somewhat noisier, but qualitatively the same.(TIF)

Protocol S1 Numerical analysis.
(NB) Click here for additional data file.

Text S1 Complete recombination.
(PDF)

Text S2 Unlinked loci.
(PDF)

Text S3 Average fixation probability.
(PDF)

Text S4 Additive effects of multiple sweeps.
(PDF)

Text S5 Fluctuations in the rate of adaptation.
(PDF)

Text S6 Variation among backgrounds and spatial variation.
(PDF)
