# Supplementary figures and images for: Limits to the Rate of Adaptive Substitution in Sexual Populations
Source: PLoS Genet. 2012 Jun 7;8(6):e1002740. doi: 10.1371/journal.pgen.1002740 (PMC3369949; doi:10.1371/journal.pgen.1002740)

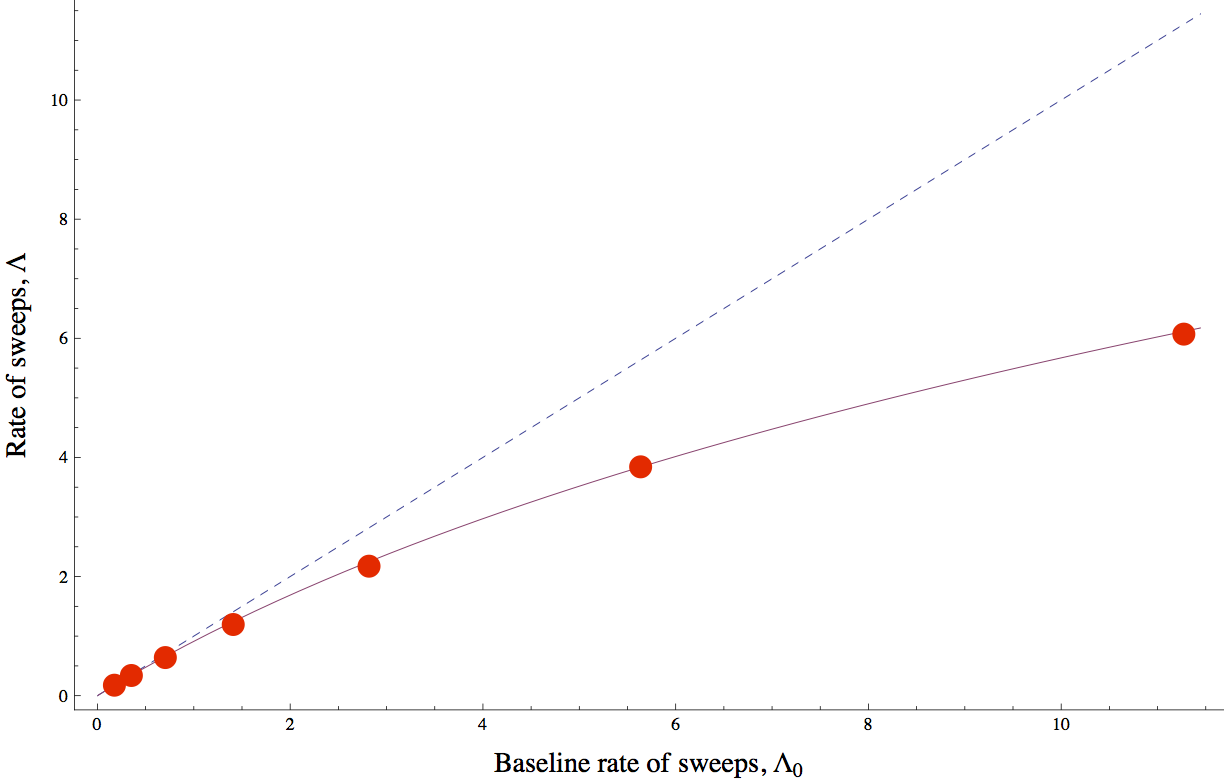

Supplement: Figure S1 — Reduction in the rate of adaptation caused by uncorrelated fitness fluctuations. The rate of selective sweeps when fitness fluctuations are uncorrelated across generations, as a function of the baseline rate in the absence of fitness fluctuations, . The dots show simulation results, the solid curve shows the theoretical prediction , and the dashed line shows . The selective advantage of mutant alleles is . For the simulations, population size is held constant at while mutation rate is varied. The points are the average rate of sweeps over 1000 simulated generations, discarding the first 200 generations. (TIF) [file pgen.1002740.s001.tif]

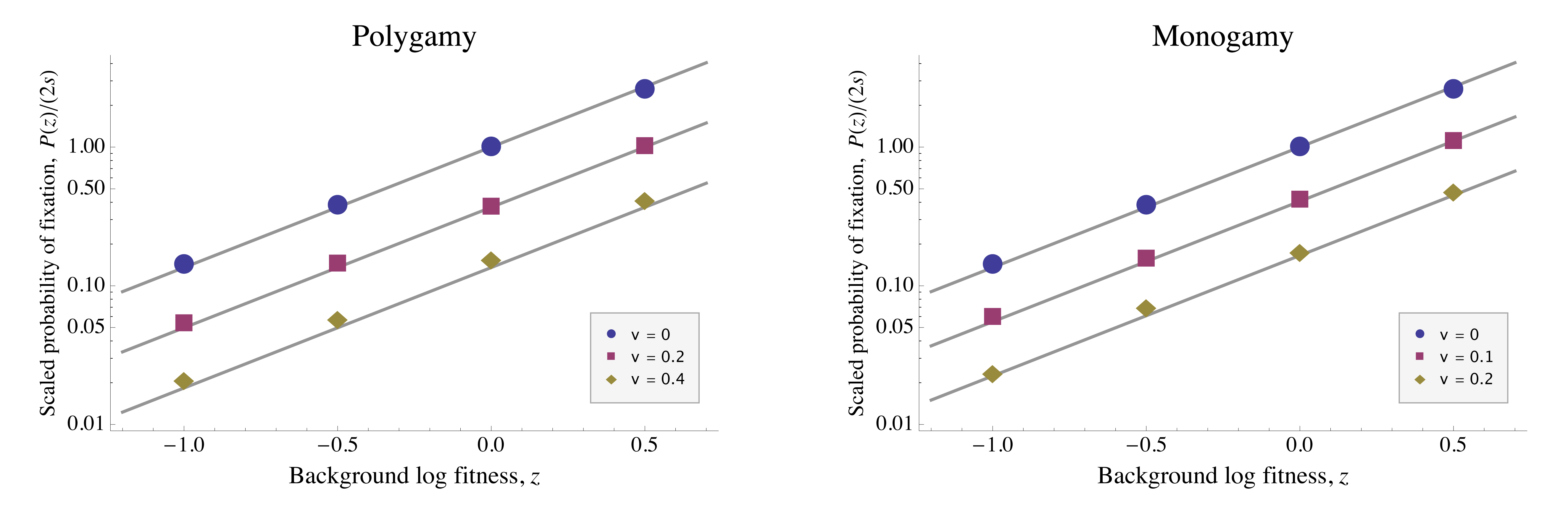

Supplement: Figure S2 — Interference among unlinked loci. The reduction in fixation probability due to inherited variation in fitness, under the infinitesimal model. The scaled fixation probability , of an allele with advantage that arises in a haploid individual with value is plotted against on a log scale. The lines show the predictions for polygamy (left panel) and for monogamy (right panel); the variance in log fitness is (left) and (right), running from top to bottom. Points show estimates from simulations of the infinitesimal model; these were run until at least 400 lineages reached a size greater than 5000 individuals, at which point they were considered fixed. Standard errors are less than the size of the points. (TIF) [file pgen.1002740.s002.tif]

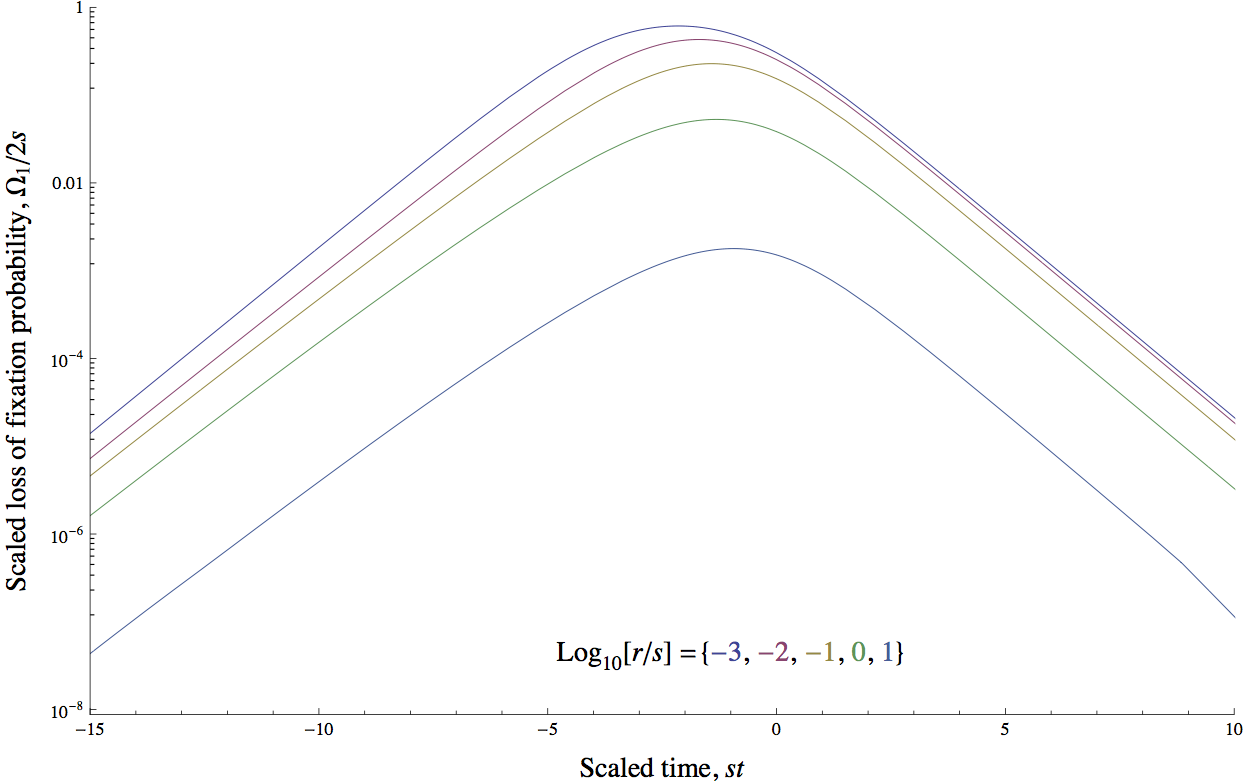

Supplement: Figure S3 — Interference caused by a single sweep over time. The scaled loss of fixation probability, , of a new allele with advantage caused by the sweep of an allele also with advantage at another locus, as a function of the scaled time between the midpoint of the sweep and the birth of the focal allele. (Negative times correspond to the focal allele arising before the interfering sweep reaches frequency 1/2.) The curves show the effect of interfering loci at scaled genetic distance (moving down). Note that for all values of the amount of interference peaks at , and falls off as away from this maximum. Note also that for , interference peaks at less than reduction in fixation probability, while for , interference depends only weakly on . is calculated numerically from Eqs. (2) and (3) . (TIF) [file pgen.1002740.s003.tif]

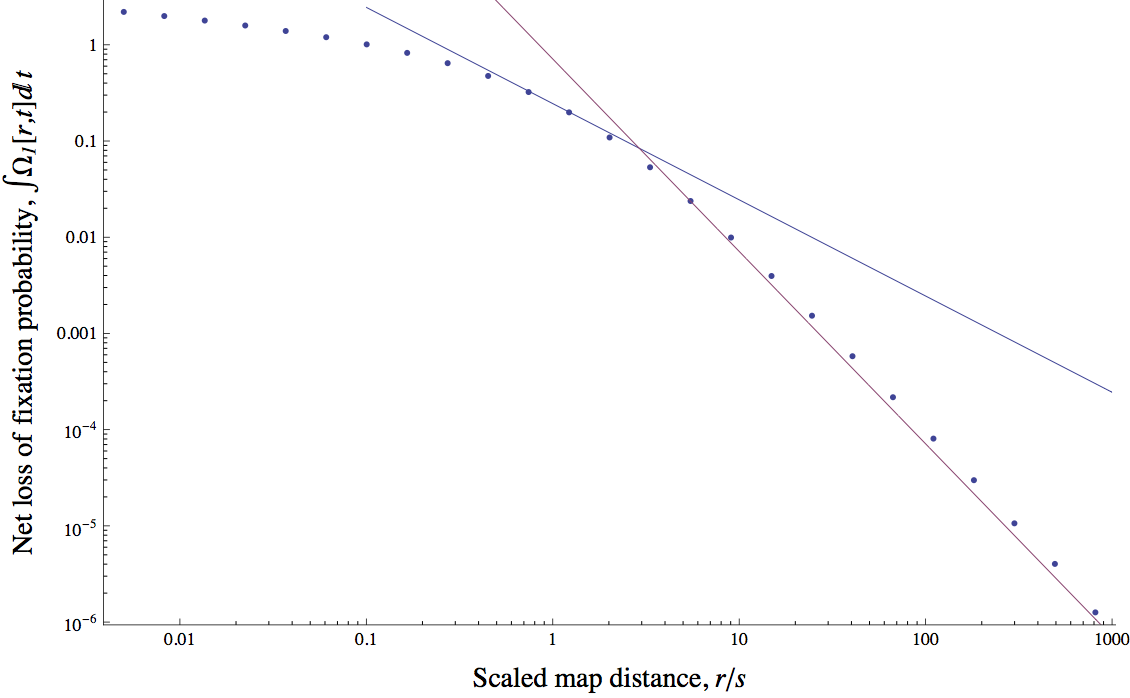

Supplement: Figure S4 — Total interference caused by a single sweep at different genetic distances. The dotted line shows the total interference caused by a selective sweep at a locus a map length away. Both the sweep and the alleles with which it is interfering have selective adavantage ; the interference then depends only on . The points are obtained by numerically solving and integrating Eqs. (2) and (3) . The solid blue line shows ; we see that the dotted line falls off faster than for , while falling off slower than for , indicating that the total interference integrated over loci (, see 4 ) is dominated by . For , the slope approaches on this log-log plot (purple line), as predicted by Robertson [54] and by our argument for unlinked loci above. (TIF) [file pgen.1002740.s004.tif]

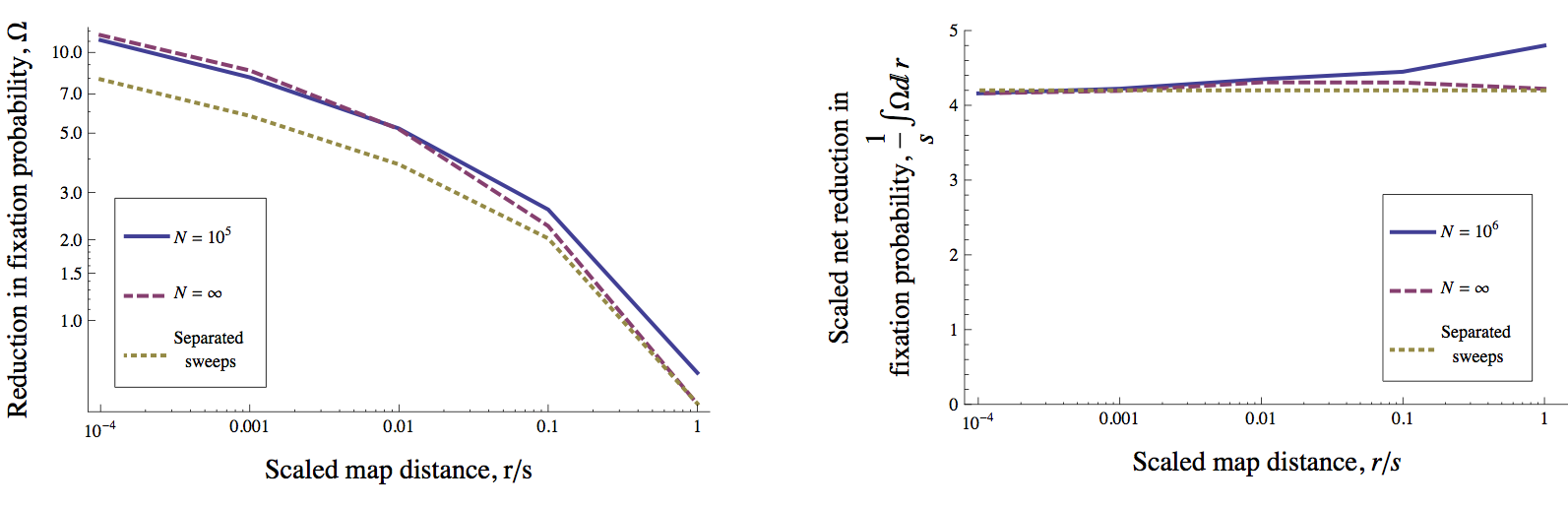

Supplement: Figure S5 — Reduction in fixation probability due to a pair of sweeps. Numerical results for the reduction in fixation probability caused by two sweeps, as a function of the distance between them. Both plots show dimensionless scaled variables, so that they are independent of the strength of selection in large populations (). Solid curves show results for a “finite population”, in which the sweeps begin in complete negative linkage disequilibrium at frequency , and then follow deterministic trajectories. Dashed curves show the results for an infinite population in which the sweeps are in linkage equilibrium. The dotted curves shows the summed effect of two sweeps that occur very far apart in time, so that there is no interaction. At all map distances, the amount of interference is close to that of two independent sweeps, even allowing for linkage disequilibrium. The curves are obtained by numerically solving and integrating Eqs. (2) and (3). Left panel: The net reduction in fixation probability at a single locus caused by two sweeps, , is plotted against the scaled map distance between the sweeps and the focal locus, which lies midway between them. is averaged over possible time intervals between the sweeps ranging from to 5; depends only weakly on this time interval, varying by less betweeen and for each of the map distances. The solid curve is for population size . Right panel: The scaled net reduction in fixation probability over the whole genome caused by a pair of simultaneous sweeps, , where the integral is over the map position of the new mutation. This is plotted against the scaled map distance between the two sweeps. The solid curve is for population size . The effects of linkage disequilibrium and interaction between the sweeps are always small, but they are largest for , when the region of the genome experiencing substantial interference from both sweeps is maximized. (At larger values of , the sweeps become approximately independent.) (TIF) [file pgen.1002740.s005.tif]

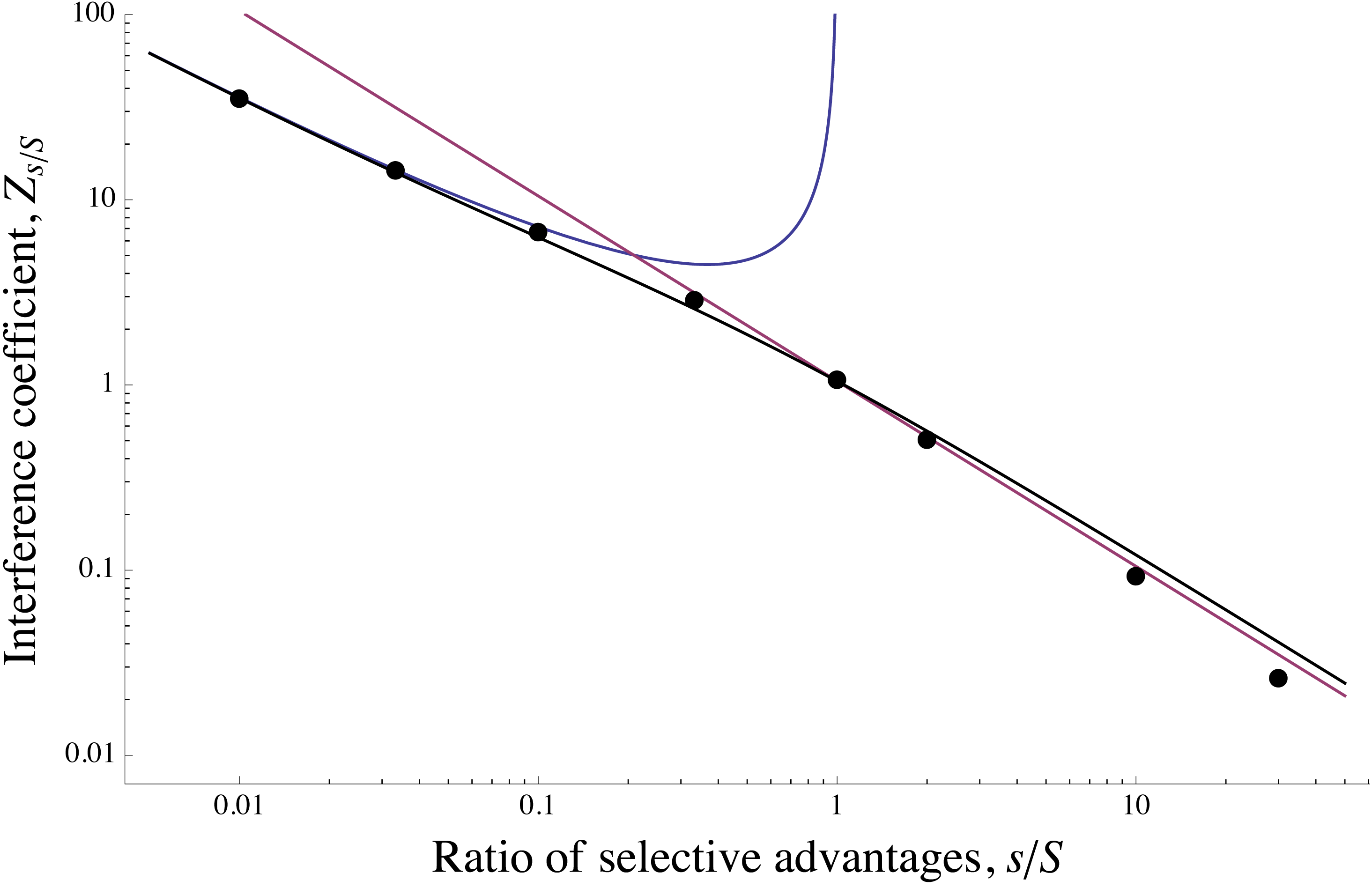

Supplement: Figure S6 — Interference coefficient . , defined in Eq. (10) , describes how much sweeps with selective coefficient interfere with alleles with selective coefficient . Points show the result of numerical integration of Eq. (6) of [55]. The blue curve shows the approximation from 4 . The purple line shows the approximation . These two approximations are valid for and , respectively. The black curve shows the combined approximation, Eq. (11) . The numerical results are expected to be overestimate (i.e., the amount of interference) for , but even so predict that the interference will typically be negligible. (TIF) [file pgen.1002740.s006.tif]

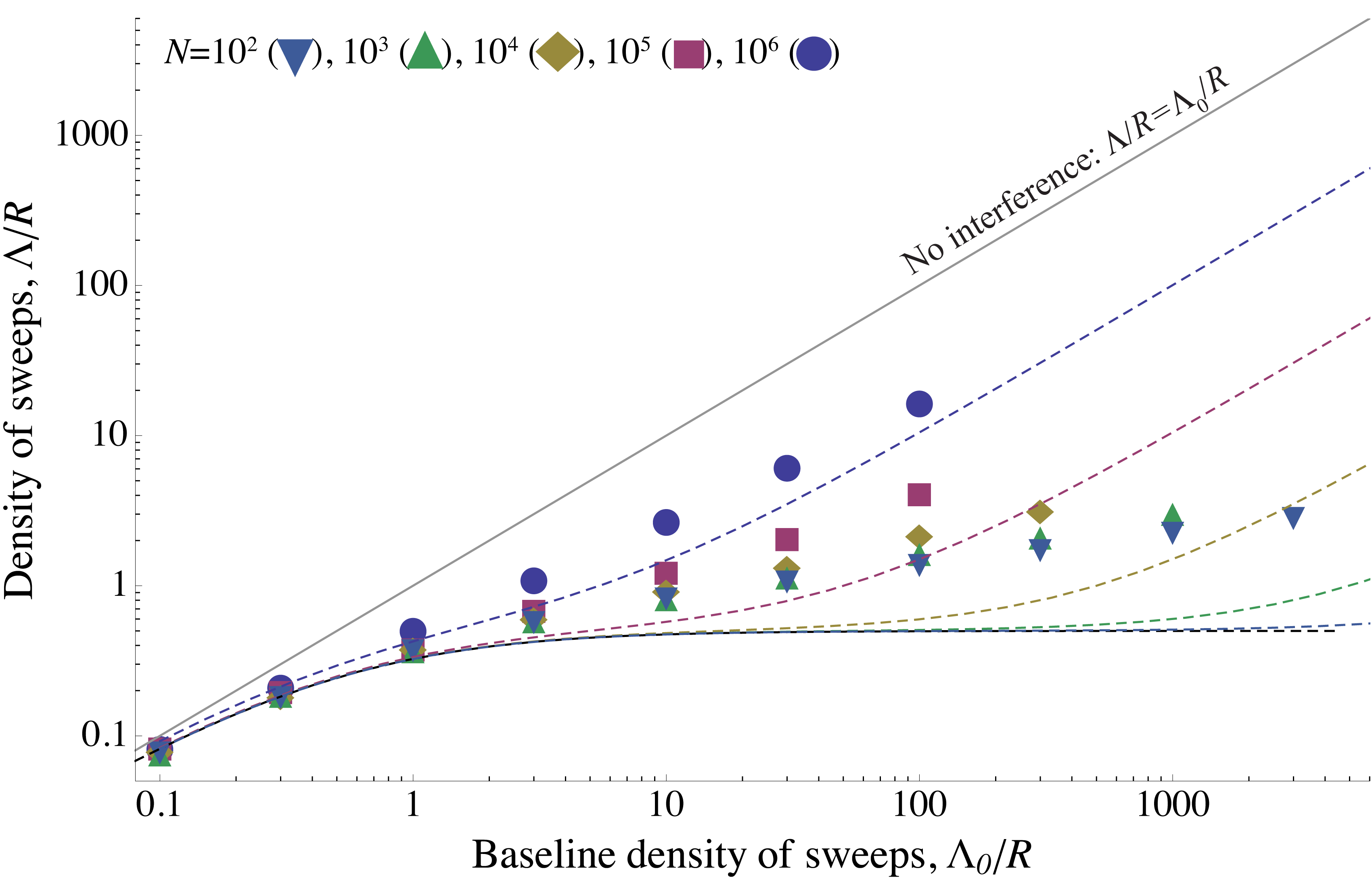

Supplement: Figure S7 — The density of sweeps as a function of the baseline density. A more detailed version of Figure 4, including the accumulation of mutations by neutral drift (combined theoretical predictions shown by dashed curves). For small populations ( for the parameters shown), drift overwhelms selection once interference becomes strong, and “adaptive” mutations become effectively neutral. In this regime, , and our scaling argument breaks down. In larger populations (), the probability of fixation remains much higher than even for strong interference. This parameter regime remains to be described analytically, but it appears that the scaling argument is still a good approximation. (TIF) [file pgen.1002740.s007.tif]

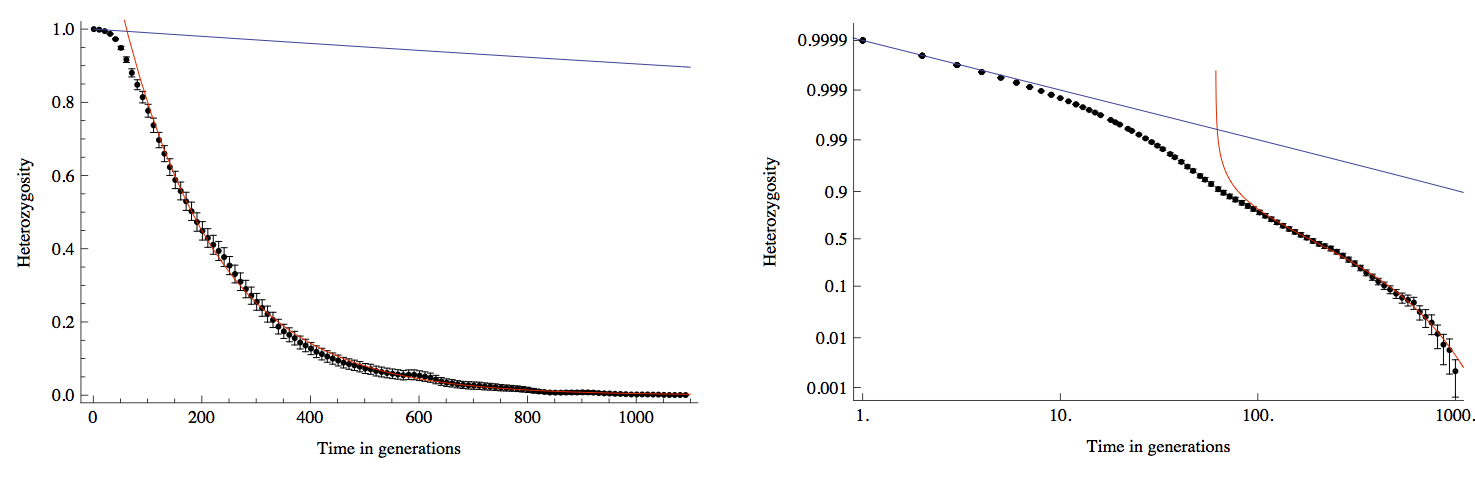

Supplement: Figure S8 — Decrease in neutral diversity over time. Decay of heterozygosity, , over time at a neutral locus, for a population in which every individual starts with a unique marker and there is no further mutation at the marker locus. The right panel shows the same data as the left, but on a log-logit scale. Initially, heterozygosity decays by neutral drift, decreasing at a rate of per generation, but then decays faster due to genetic draft. Since the stochasticity introduced by genetic draft has different strengths over different time scales, it cannot be fully described by adjusting a single “effective population size.” Black dots are averages over 100 simulation runs, with error bars showing the standard error. The blue curves show the heterozygosity expected for a population evolving neutrally in continuous time, . The red curves are a fit to the simulation data for , when the heterozygosity has approached its long-term rate of decrease: , where is an offset to account for the initial slow decrease in . The inferred value is insensitive to the exact fitting method used. Parameters are as in Figure 6, with and beneficial mutation rate , corresponding to . (The curves for other values of are qualitatively the same.) (TIF) [file pgen.1002740.s008.tif]

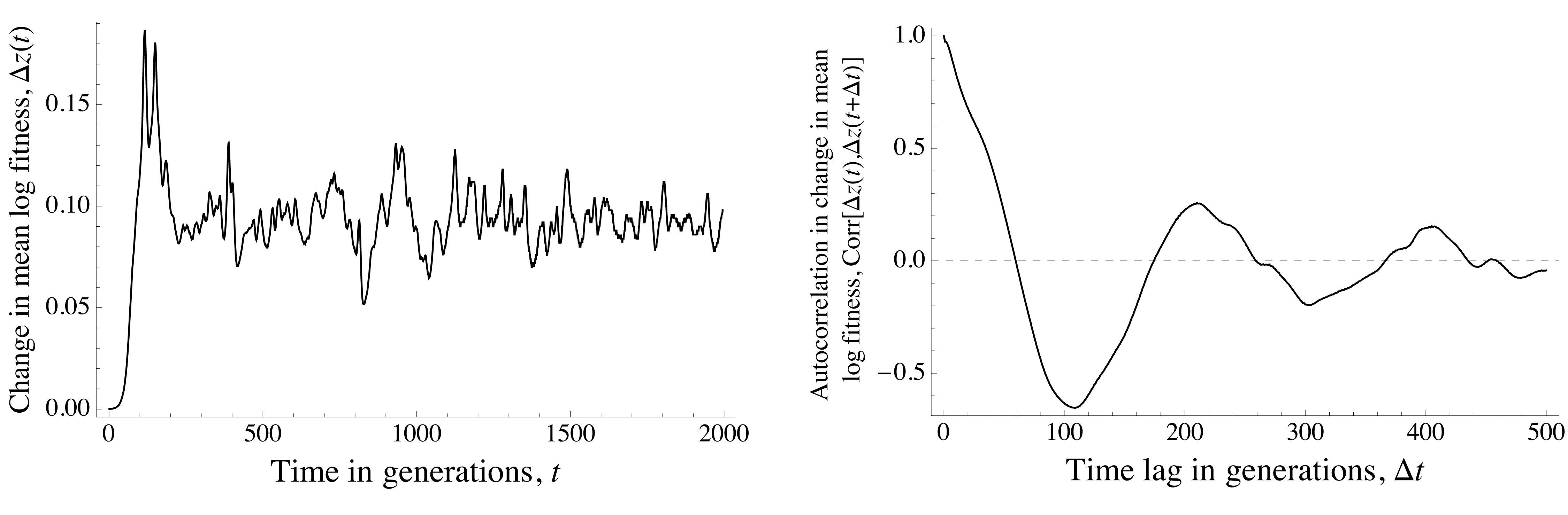

Supplement: Figure S9 — Variation in rate of increase of mean fitness. The increase in mean log fitness per generation, (left panel), and the auto-correlation function (right panel) for a simulated population. is negatively auto-correlated on the time scale over which alleles go from a few copies to the frequency at which they cause the most interference. The population was initially monomorphic, and thus starts low, then spikes as the first wave of mutations reach intermediate frequencies. This wave then strongly interferes with new mutations, causing a later decrease in ; etc. The population parameters are as in Figure 5, with . Data in the left panel are averaged over a 5-generation window. Excluding the first 500 generations leaves the auto-correlation shown in the right panel somewhat noisier, but qualitatively the same. (TIF) [file pgen.1002740.s009.tif]
