# Supplementary material for: Limits to the Rate of Adaptive Substitution in Sexual Populations
Source: PLoS Genet. 2012 Jun 7;8(6):e1002740. doi: 10.1371/journal.pgen.1002740 (PMC3369949; doi:10.1371/journal.pgen.1002740)
Supplement: Text S1 — Complete recombination. (PDF) [file pgen.1002740.s011.pdf]

Text S1 Complete recombination

We checked the effect of fitness variance in the absence of linkage disequilibrium by simulating a large number of concurrent selective sweeps. The population is represented by two lists: the numbers of copies of rare favorable alleles, $\underline{n}$, and the frequencies of common alleles, $\underline{p}$. Whenever an allele rises above 100 copies, it is assumed to increase deterministically: therefore, it is deleted from the first list, and its frequency added to the second list. In each generation, a Poisson number of new mutations is added, with expectation NU, and each in a single copy. The common allele frequencies $\underline{p}$ evolve deterministically, so that $p_i \to \frac{1+s}{1+sp_i} p_i$ every generation. The key step is to draw the number of copies of each rare allele. For each locus, the distribution of frequencies of genetic backgrounds is calculated. for example, a gene at locus 1 will find itself on background $000\ldots0$ with probability $q_2 \ldots q_k$; with k loci, there are 2^{k-1} possible backgrounds for each locus. However, we only need to track the numbers of each fitness class, defined by the number of favorable alleles it carries. The n_i copies at locus i are distributed over the possible fitness classes with multinomial probability, and for each fitness class, a Poisson number of offspring is drawn with the appropriate expectation. The rate of sweeps is measured after 200 generations. Figure S1 shows a close match between the simulated and predicted rate of accumulation of favorable alleles.
