# Supplementary material for: Limits to the Rate of Adaptive Substitution in Sexual Populations
Source: PLoS Genet. 2012 Jun 7;8(6):e1002740. doi: 10.1371/journal.pgen.1002740 (PMC3369949; doi:10.1371/journal.pgen.1002740)
Supplement: Text S2 — Unlinked loci. (PDF) [file pgen.1002740.s012.pdf]

Text S2 Unlinked loci

An additive trait, z, is determined by a very large number of unlinked loci. Under the infinitesimal model, a pair of individuals with trait values y, z produce offspring with a normal distribution of trait values, with mean $(y+z)/2$ and variance $v/2$. The population is at linkage equilibrium, with variance v. The trait distribution in generation t, $\psi_t(z)$, has variance v and mean $\bar{z} = v(t-1/2)$. The offset of 1/2 is arbitrary and is chosen to simplify the form of the expected number of offspring of a haploid individual, which is $e^{z-\bar{z}-v/2} = e^{z-vt}$. This is normalized such that the mean over z is always 1, as required. (Here, and in the following derivation, we will use the relation $E\left[e^{\alpha z}\right] = e^{\alpha m+\alpha^2 v/2}$, where the mean and variance of the distribution are m, v, respectively. For α=1, the normalization factor $e^{-v/2}$ ensures that $E\left[e^{z-\bar{z}-v/2}\right] = 1$).

A single copy of a favorable allele, with selective advantage $s > 0$, arises at time $t = 0$ on a genetic background z. Note that the individual with the allele thus has fitness $(1+s)e^z$. The allele's chance of fixation is $P_0(z)$, and of loss, $Q_0(z) = 1 - P_0(z)$. Since the distribution of z shifts by v in each generation, we must have $P_t(z) = P_0(z - vt)$.

The fixation probability depends on the distribution of offspring number, and so is sensitive to the choice of life cycle. For example, if haploid individuals had a chance θ of surviving to breed, and then had a Poisson number of offspring with expectation $1/\theta$, the variance of offspring number would be $1/\theta$, and fixation probability would be reduced by a factor θ. Thus, it is simpler to assume fertility selection, in which all differences in fitness are due to differences in the mean number of offspring produced. We consider two models: polygamous and monogamous. In the first model, an individual with trait value z produces a Poisson number of offspring with expectation e^z, each with a different mate, with the mates' trait values drawn from the distribution $\psi(y)e^y$. In the second model, mated pairs with trait values $\{y, z\}$ are chosen with probability $\psi(y)\psi(z)$ and produce a Poisson number of offspring with expectation e^{y+z}. This generates substantially more random drift, because the random value of an individual's mate affects all its offspring, rather than just one.

Polygamy

The probability of loss is given by averaging over the distribution of offspring number, j, over the distribution of trait values of the mates, y, and finally, over the distribution of trait values of offspring,

x:

$$
\begin{aligned}
Q_0(z) =& \sum_{j=0}^{\infty} \frac{e^{-\lambda}\lambda^j}{j!} \left(\int_{-\infty}^{\infty} \psi(y) e^y \int_{-\infty}^{\infty} \phi(x|y,z) Q_1(x) dx dy \right)^j \\
=& \exp\left(-\lambda \int_{-\infty}^{\infty} \psi(y) e^y \int_{-\infty}^{\infty} \phi(x|y,z) P_1(x) dx dy \right),
\end{aligned}
$$

where $\lambda = (1+s)e^z$ and the distribution of mate values, with density $\psi(y)e^y$, is a Gaussian with mean $v/2$ and variance v. $\phi(x|z,y)$ is the distribution of offspring trait values, which is Gaussian with mean $(z+y)/2$ and variance $v/2$. Assuming that $s \ll 1$, $P = \mathcal{O}(s)$, and substituting for λ, we have:

$$
\begin{aligned}
P_0(z) =& (1+s) \int_{-\infty}^{\infty} \psi(y) e^{y+z} \int_{-\infty}^{\infty} \phi(x|y,z) P_1(x) dx dy \\
& - 1/2 \left(\int_{-\infty}^{\infty} \psi(y) e^{y+z} \int_{-\infty}^{\infty} \phi(x|y,z) P_1(x) dx dy \right)^2 + \mathcal{O}\left(s^3\right). \qquad (16)
\end{aligned}
$$

Note that this expansion breaks down for sufficiently large z – the following derivation only applies to populations in which no more than a negligible number of individuals have a trait value z high enough such that $P_0(z)$ approaches 1. We will verify below that this includes realistically-sized populations.

Eq. (16) can be rewritten as:

$$
P_0(z) = (1+s)\tilde{P}_0(z) - \frac{1}{2}\tilde{P}_0(z)^2 + \mathcal{O}\left(s^3\right), \qquad (17)
$$

where $\tilde{P}_0(z)$ is given by

$$
\begin{aligned}
\tilde{P}_0(z) \equiv& e^z \int_{-\infty}^{\infty} \int_{-\infty}^{\infty} \psi(y) e^y \phi(x|y,z) P_1(x) dx dy \\
=& \sqrt{\frac{2}{3\pi v}} e^z \int_{-\infty}^{\infty} \exp\left[-\frac{2}{3v} \left(x - \frac{2z-3v}{4} \right)^2 \right] P_0(x) dx. \qquad (18)
\end{aligned}
$$

(In the last line we have used that $P_1(x) = P_0(x-v)$.) We can think of $(1+s)\tilde{P}_0(z)$ as the expected long-term contribution of an individual with value z - that is, its reproductive value [49].

Notice that Eq. (17) implies that $P_0(z) = \tilde{P}_0(z) + \mathcal{O}\left(s^2\right)$. In other words, to lowest order in s, P_0 is an eigenfunction of the "tilde" operator defined in Eq. (18). This operator resembles a generalized Weierstrass transform or Gaussian filter, which have exponentials as eigenfunctions. This suggests that

we guess that $P_0(z)$ can be approximated by an exponential: $P_0(z) = 2sCe^{\alpha z} + \mathcal{O}\left(s^2\right)$, where C and α do not depend on z. Eq. (18) then gives $\tilde{P}_0(z) = 2sC\exp\left(z + \alpha\left(\frac{z}{2} - \frac{3v}{4}\right) + \frac{\alpha^2}{2}\frac{3v}{4}\right)$. Equating our guess for $P_0(z)$ and our expression for $\tilde{P}_0(z)$, we see that we must have $\alpha = 2 + \mathcal{O}(s)$ for consistency.

To determine the constant C, take the expectation of Eq. (17) over the distribution of z, $\psi(z)$. Let the average fixation probability at time t be $E\left[P_t[z]\right] = \bar{P}$. (Since $\bar{P}$ stays the same across generations, we can drop the subscript.) Taking the expectation of Eq. (18) over log fitness z, we find that $E\left[\tilde{P}_0\right] = E\left[P_0\right]$. Using this, we can simplify the expectation of Eq. (17) to obtain, to leading order in s,

$$2s\bar{P} = \overline{P(z)^2} = \bar{P}^2 + \mathrm{Var}\left[P\right]. \tag{19}$$

This is a discrete-time version of Eq. (3), above. Since we know that $P(z)$ has the form $2sCe^{2z}$, and z has a normal distribution with mean $-\frac{v}{2}$, variance v, we have that $\bar{P} = 2sCe^{v}$ and $\overline{P(z)^2} = 4s^2C^2e^{6v}$. Eq. (19) thus implies that $C = e^{-5v}$, so $\bar{P} = 2se^{-4v} + \mathcal{O}\left(s^2\right)$. (Note that $P(z) = 2se^{2z-5v}$ does not approach 1 until $z \sim \frac{5v}{2} - \log(2s)$, a trait value too large to be found except in impossibly large populations.) Recall that the variance in log fitness is equal to the rate of change in mean log fitness, $v = NU\left\langle \bar{P}\log(1+s)\right\rangle$, where the brackets denote the expectation over s. Inserting our expression for $\bar{P}$, this becomes $v = 2NU\left\langle s\log(1+s)\right\rangle e^{-4v} = v_0e^{-4v}$, where $v_0 = 2NU\left\langle s\log(1+s)\right\rangle$ is baseline rate of increase in mean log fitness in the absence of interference. This implicit equation for v has the solution $v = \frac{1}{4}\mathfrak{W}\left(4v_0\right)$. This implies that v increases only logarithmically with v_0 for $v_0 \gg 1$.

Monogamy

The derivation for this case proceeds in the same way, but with the order of events changed. The probability of loss is given by averaging over the randomly chosen mate, with trait value y, then over the distribution of offspring number, j, and then over the distribution of trait values of offspring, x:

$$\begin{aligned} Q_0(z) &= \int_{-\infty}^{\infty}\psi(y)\sum_{j=0}^{\infty}e^{-\lambda}\frac{\lambda^j}{j!}\left(\int_{-\infty}^{\infty}\phi(x|y,z)Q_1(x)dx\right)^j dy \\ &= \int_{-\infty}^{\infty}\psi(y)\exp\left(-\lambda\int_{-\infty}^{\infty}\phi(x|y,z)P_1(x)dx\right)dy \end{aligned}$$

where $\lambda = (1+s)e^{y+z}$. Assuming that $s \ll 1$, $P = \mathcal{O}(s)$, and substituting for λ, we have:

$$P_0(z) = (1+s)\tilde{P}_0(z) - \frac{1}{2}R_0(z) + \mathcal{O}\left(s^3\right), \tag{20}$$

where $R_0(z)$ is defined as

$$R_0(z) = \int_{-\infty}^{\infty} \psi(y) \left(e^{y+z} \int_{-\infty}^{\infty} \phi(x|y,z) P_1(x) dx \right)^2 dy.$$

The first term on the right-hand side of Eq. (20) is proportional to $\tilde{P}_0(z)$, which is defined as before. By the same argument as above, $P_0(z) = \tilde{P}_0(z) + o(s)$, so if we guess that $P_0(z){=}2sCe^{\alpha z}$, we find that for consistency we must again have $\alpha = 2$. Taking expectations of both sides of Eq. (20) over the distribution of log fitness z and again using $P_1(x) = P_0(x-v)$ gives the constant $C = e^{-9v}$. Thus $P(z) = 2se^{2z-9v}$, and again we see that our assumption that $P(z) \ll 1$ is valid for any biological value of z. Taking the expectation over the distribution of z gives the average fixation probability, $\bar{P} = 2se^{-8v}$. The rate of increase of mean log fitness is therefore $v = \frac{1}{8}\mathfrak{W}(8v_0)$.

Simulations

Figure S2 compares these predictions with C simulations in which a single individual carrying an allele with advantage $s = 0.05$ is introduced in a single background with value z, and then reproduces as a branching process according to the infinitesimal model (i.e., the distribution of log fitnesses is $\psi(y)e^y$ for mates, $\phi(x|y,z)$ for offspring, etc.). The simulation results agree well with the predictions. Lineages were classified as successfully sweeping once they grew to more than 5000 individuals. (Since $5000\bar{P} > 20$ for all simulated parameters, essentially all lineages that reach size 5000 should fix.) The left panel is for the polygamous model, with variance $v = 0, 0.2, 0.4$, while the right panel is for the monogamous model, with $v = 0, 0.1, 0.2$; recall that z is distributed with mean $-v/2$, variance v. When there is no variance in the population ($v = 0$), the model is that an individual with value z mates with another individual with value $y = 0$, to produce offspring with $x = z/2$; there is no difference between monogamy and polygamy in this case. Note that the more detailed simulations of individuals with long linear chromosomes shown in Figure 3 also confirm the analytical results for the case of polygamy.
