# Supplementary material for: Limits to the Rate of Adaptive Substitution in Sexual Populations
Source: PLoS Genet. 2012 Jun 7;8(6):e1002740. doi: 10.1371/journal.pgen.1002740 (PMC3369949; doi:10.1371/journal.pgen.1002740)
Supplement: Text S3 — Average fixation probability. (PDF) [file pgen.1002740.s013.pdf]

Text S3 Average fixation probability

We wish to find the rate of change of the average probability of fixation of a focal allele,

$$\partial_t \bar{P} = \sum_{\underline{X}} g\left(\underline{X}\right) \partial_t P\left(\underline{X}\right) + \sum_{\underline{X}} \partial_t g\left(\underline{X}\right) P\left(\underline{X}\right). \tag{21}$$

The first sum is given by the average of Eq. (2), weighted by genotype frequencies:

$$-\sum_{\underline{X}} g\left(\underline{X}\right) \partial_t P\left(\underline{X}\right) = s\bar{P} + \sum_{\underline{X}} g\left(\underline{X}\right) S\left(\underline{X}\right) P\left(\underline{X}\right) + \sum_{\underline{X},\underline{Y}} g\left(\underline{X}\right) r\left(\underline{X},\underline{Y}\right) \left(P\left(\underline{Y}\right) - P\left(\underline{X}\right)\right) - \frac{1}{2} \sum_{\underline{X}} g\left(\underline{X}\right) P\left(\underline{X}\right)^2 \tag{22}$$

To calculate the second sum in Eq. (21), we require the rate of change of background frequencies:

$$\partial_t g\left(\underline{X}\right) = S\left(\underline{X}\right) g\left(\underline{X}\right) + \frac{1}{2} \sum_{\underline{Y}} \left(g\left(\underline{Y}\right) R\left(\underline{Y},\underline{X}\right) - g\left(\underline{X}\right) R\left(\underline{X},\underline{Y}\right)\right), \tag{23}$$

where $R\left(\underline{X},\underline{Y}\right)$ is the rate at which individuals with genotype $\underline{X}$ recombine to form individuals with genotype $\underline{Y}$. (The factor of 1/2 in Eq. (23) is necessary because each recombination event involves two parents recombining to form two offspring.) Note that unlike $r\left(\underline{X},\underline{Y}\right)$ (the rate at which recombination moves the focal allele from background $\underline{X}$ to background $\underline{Y}$), the definition of $R\left(\underline{X},\underline{Y}\right)$ does not involve the focal allele, and in general $r\left(\underline{X},\underline{Y}\right) \neq \frac{1}{2} R\left(\underline{X},\underline{Y}\right)$. However, it is true that $\sum_{\underline{X}} g\left(\underline{X}\right) r\left(\underline{X},\underline{Y}\right) = \frac{1}{2} \sum_{\underline{X}} g\left(\underline{X}\right) R\left(\underline{X},\underline{Y}\right)$ for all $\underline{Y}$, since both sides are expressions for the total rate of recombination events producing offspring with genotype $\underline{Y}$. Thus, when we substitute Eqs. (22) and (23) into Eq. (21), we find that the terms involving S and r cancel, leaving

$$-\frac{\partial \bar{P}}{\partial t} = s\bar{P} - \frac{1}{2} \sum_{\underline{X}} g\left(\underline{X}\right) P\left(\underline{X}\right)^2. \tag{24}$$

Rewriting the second term in terms of the mean and variance of $P\left(\underline{X}\right)$, we obtain Eq. (3).
