# Supplementary material for: Limits to the Rate of Adaptive Substitution in Sexual Populations
Source: PLoS Genet. 2012 Jun 7;8(6):e1002740. doi: 10.1371/journal.pgen.1002740 (PMC3369949; doi:10.1371/journal.pgen.1002740)
Supplement: Text S4 — Additive effects of multiple sweeps. (PDF) [file pgen.1002740.s014.pdf]

Text S4 Additive effects of multiple sweeps

Effect of multiple catastrophes

We wish to find the probability of fixation $\bar{P}$ of a new allele providing advantage s when there are ongoing sweeps at linked loci, averaged over possible patterns of sweeps and genetic backgrounds. To do so, we will first consider a related problem: suppose that a sequence of instantaneous "catastrophes" occurs at times $t_1 < t_2 < \ldots < t_n$, and that the probability that a mutant individual survives the catastrophe at time t_i is given by w_i. Then the probability that a mutant allele that arises at time t will fix is [79]

$$P(t) = 2s \times \begin{cases} \left(1 + x_1 \left(-1 + \frac{1}{w_1}\left(1 + y_2\left(-1 + \frac{1}{w_2}(\ldots)\right)\right)\right)\right)^{-1} & \text{for } t < t_1 \\ \left(1 + x_{i-1} \left(-1 + \frac{1}{w_{i-1}}\left(1 + y_i\left(-1 + \frac{1}{w_i}(\ldots)\right)\right)\right)\right)^{-1} & \text{for } t_i < t < t_{i+1} \\ 1 & \text{for } t_n < t, \end{cases} \tag{25}$$

where $x_i = \exp\left[-s\left(t_i - t\right)\right]$ and $y_i = \exp\left[-s\left(t_i - t_{i-1}\right)\right]$.

We can find the net reduction in fixation probability, integrated over randomly timed catastrophes, as follows, with $c_i = -1 + \frac{1}{w_1}\left(1 + y_{i+1}\left(-1 + \frac{1}{w_{i+1}}(\ldots)\right)\right)$:

$$\begin{aligned}
\Omega_n \equiv & \int_{-\infty}^{\infty} (2s - P)\, dt \\
= & 2s \int_{-\infty}^{t_1} \frac{x_1 c_1}{1 + x_1 c_1} dt + 2s \int_{t_1}^{t_2} \frac{x_2 c_2}{1 + x_2 c_2}\, dt + \ldots + 2s \int_{t_{n-1}}^{t_n} \frac{x_n\left(-1 + \frac{1}{w_n}\right)}{1 + x_n\left(-1 + \frac{1}{w_n}\right)}\, dt \\
= & 2\left(\log\left[1 + c_1\right] + \log\left[\frac{1 + c_2}{1 + y_2 c_2}\right] + \ldots \log\left[\frac{1/w_n}{1 + y_n\left(-1 + \frac{1}{w_n}\right)}\right]\right) \\
= & 2\log\left[\left(1 + c_1\right)\frac{1 + c_2}{w_1\left(1 + c_1\right)} \cdots \frac{1/w_n}{w_{n-1}\left(1 + c_{n-1}\right)}\right] \\
= & 2\log\left[\frac{1}{w_1 w_2 \ldots \mathrm{w}_n}\right]
\end{aligned}$$

(Above we have used that $1 + y_i c_i = w_{i-1}\left(1 + c_{i-1}\right)$. This is just the sum of effects of each individual catastrophe. Therefore, the expected net reduction in fixation probability, $E\left[\Omega\right] \equiv 2s - \bar{P}$, is equal to the rate of catastrophes, Λ, multiplied by the expected effect of a single catastrophe, $E\left[\Omega\right] = 2\Lambda E\left[\log\left[\frac{1}{w}\right]\right] = \Lambda E\left[\Omega_1\right]$.

We can now return to the problem of interfering sweeps. If all the interfering sweeps have selective advantage $S_i \gg s$, then their effect is nearly the same as the series of catastrophes considered above, with a sweep at distance r from the focal allele having effect $w = 1 - (s/S)^{r/S}$ [79]. Assuming that the sweeps with advantage S occur at rate Λ_S and are scattered uniformly over a linear map of length $R \gg S$ (i.e., r is distributed approximately uniformly), the average amount of interference is approximately:

$$E[\Omega] = \frac{4}{R} \int dS \, \Lambda_S \int_0^\infty dr \log \left[\frac{1}{1 - (s/S)^{r/S}} \right]. \tag{26}$$

(Note that there is a factor of 2 in Eq. (26) arising from the fact that sweeps cause interference to both upstream and downstream loci on the chromosome.) If all interfering sweeps have the same selective advantage S and occur at rate Λ, this simplifies to

$$E[\Omega] = \frac{2\pi^2 S \Lambda}{3R \log[S/s]}. \tag{27}$$

Thus the expected fixation probability of the focal allele is

$$\bar{P} = 2s \left(1 - \frac{\pi^2 S \Lambda}{3Rs \log[S/s]} \right). \tag{28}$$

This is a proof of the linearity result in [79]: that paper only established the threshold at which alleles become effectively neutral and showed the linear relation between $\bar{P}$ and Λ numerically.

Numerical analysis

In the main text, we instead focus on the case $S \to s$ in which all alleles have the same effect. In this case, the above proof of linearity no longer applies, because the interference caused by a sweep can no longer be approximated by an instantaneous catastrophe. Instead, the amount of interference grows and decays in time approximately exponentially with st, peaking at $\sim 1/s$ generations before the sweep reaches frequency 1/2 (Figure S3). However, the numerical calculations below show that for small numbers of interfering sweeps the effects nearly add. Thus, as long as no more than a few sweeps are typically occurring nearby on the chromosome ($\Lambda/R \lesssim 1$), we can continue to make the approximation that their

effects add. Doing so, we can write the total expected interference experienced by a new allele as

$$E[\Omega] = \Lambda \int_0^R dr \int_{-\infty}^{\infty} dt\, \Omega_1(r,t) p(r), \tag{29}$$

where $p(r)$ is the probability density for the map length between the focal allele and a sweep; assuming that both are distributed uniformly, it is $p(r) = 2(1 - r/R)/R$ as long as $r \ll 1/2$ (the value at which the genetic distance saturates).

$\Omega_1(r,t)$ can be found by numerically solving Eq. (5) from [55], after which the integration in Eq. (29) can also be performed numerically. The integral over r is dominated by $r \sim s$ (see Figure S4), so for $R \gg s$ far-away sweeps make little contribution (besides the net effect of unlinked sweeps discussed in the main text), and we can take R to infinity in the integral. In this case, $p(r)$ reduces to a uniform distribution, and Eq. (29) evaluates to

$$E[\Omega] \approx \frac{4Z\Lambda s}{R}, \tag{30}$$

where $Z \approx 1.05$. Note that Z does not depend on any of the population parameters. Since $\bar{P} = 2s - E[\Omega]$, Eq. (6) immediately follows.

Checking the additive approximation

We can check the assumption of additivity by numerically solving Eq. (2) for the fixation probability. To do this, we must know the frequencies of the selected genetic backgrounds, $g\left(\underline{X}\right)$. (Note that the number of possible backgrounds grows exponentially with the number of polymorphic loci, so it is impractical to do this for more than a few interfering sweeps). We assume that each interfering sweep begins with a single copy in a wild-type individual, and then follows the deterministic trajectory (given by Eq. (23)). (Note that after this initialization, we allow for recombinant genetic backgrounds to be present at arbitrarily low frequencies.) The deterministic approximation underestimates the typical initial rate of increase while the sweeping alleles are at frequencies $\lesssim 1/(Ns)$ [62], but the contribution of this very early part of the trajectory to the total interference is negligible. The sweeping alleles will appear at appreciable frequency after a time $t^* \sim \frac{1}{s}\log(Ns)$. The sweeps will initially be in complete negative linkage disequilibrium; the extent of linkage disequilibrium that remains when they reach high frequency, and hence the extent to which they interfere with each other, depends on $rt^* = \left(\frac{r}{s}\right)\log(Ns)$. Thus, population size does have an indirect influence on the rate of adaptation, by influencing the extent of linkage disequilibrium

between competing sweeps. However, numerical calculations show that in large populations, linkage disequilibrium between sweeps will typically decay to a negligible value before they reach frequencies at which they appreciably interfere with new mutations. The total interference caused by two overlapping sweeps therefore depends only weakly on the initial linkage disequilibrium between them, and in fact is close to the interference caused by two isolated (non-overlapping) sweeps. The numerical results shown in Figure S5 show that pairs of sweeps do typically have a stronger effect than expected from their individual effects, but that this interaction is generally small compared to the total reduction in fixation probability.

Distribution of selective coefficients

We can repeat the analysis leading to Eq. (30) for a distribution $\rho(S)$ of selective advantages of the interfering sweeps. We can use the generalization of Eqs. (26) and (29):

$$E[\Omega] = \frac{2}{R} \int dS \, \Lambda_S \int_0^\infty dr \int_{-\infty}^\infty dt \, \Omega_1(r, t, S), \tag{31}$$

where we have assumed that nearly all sweeps have $S \ll R$, so that $p(r) \approx 2/R$. Eq. (4) and the scaling argument in the main text imply that the total interference caused by a sweep must have the form

$$\int_0^\infty dr \int_{-\infty}^\infty dt \, \Omega_1(r, t, S) = 2s Z_{S/s}, \tag{32}$$

where $Z_{s/S}$ depends only on the ratio of the selective coefficients; see [55] for a more detailed discussion. $Z_{S/s}$ can be evaluated numerically by integrating Eq. (6) of [55]. Simply gluing together the approximations in Eqs. (27) and (30) suggests that we approximate $Z_{s/S}$ by Eq. (11), which does indeed fit the numerical analysis well for $S \not\ll s$ – see Figure S6. (The numerical analysis fails in any case for $S \ll s$, since [55] assumes that the interfering sweep is not affected by the focal sweep.)

For the specific case considered in the main text of mutations with an exponential distribution of effects, $\rho(s) = e^{-s/\langle s \rangle} / \langle s \rangle$, most alleles that would fix in the absence of interference have selective advantages $s \sim \langle s \rangle$, as do most of the alleles that they experience interference from (see Figures 7 and 8). Thus, since S/s will rarely be greater than ≈ 3 for the relevant pairs of alleles, we can make the simplifying approximation that the logarithmic factor in Eq. (11) is roughly constant, and therefore $Z_{s/S} \approx S/s$.

With this simplification, Eq. (10) for the probability of fixation becomes:

$$\bar{P}(s) \approx 2s - \frac{4NU}{R} \int dS\, S\bar{P}(S)\rho(S)$$
$$= 2s - 4NU\hat{s}\left\langle \bar{P} \right\rangle /R, \tag{33}$$

where $\hat{s}$ is the mean selective advantage of alleles that successfully sweep. Averaging over s and rearranging terms, the mean probability of fixation is

$$\left\langle \bar{P} \right\rangle \approx \frac{2\left\langle s \right\rangle}{1 + 4NU\hat{s}/R}. \tag{34}$$

$\hat{s} = 2\left\langle s \right\rangle$ when interference is weak, and does not increase much even for strong interference (Figure 7); substituting in this value, we have

$$\left\langle \bar{P} \right\rangle \approx \frac{2\left\langle s \right\rangle}{1 + 4\Lambda_0/R}, \tag{35}$$

where $\Lambda_0 = 2NU\left\langle s \right\rangle$ is the rate of sweeps in the absence of interference.

In Figure 8, we consider populations where interference from unlinked loci is substantial ($v > 1/4$), and consider values of s large enough that the saturation at 1 must be taken into account; we therefore use the more detailed equation

$$\bar{P}(s) \approx 1 - \exp\left[-2(s - s^*)e^{-4v}\right], \tag{36}$$

where $s^* = 4\Lambda\left\langle s \right\rangle /R$.
