# Supplementary material for: Limits to the Rate of Adaptive Substitution in Sexual Populations
Source: PLoS Genet. 2012 Jun 7;8(6):e1002740. doi: 10.1371/journal.pgen.1002740 (PMC3369949; doi:10.1371/journal.pgen.1002740)
Supplement: Text S5 — Fluctuations in the rate of adaptation. (PDF) [file pgen.1002740.s015.pdf]

Text S5 Fluctuations in the rate of adaptation

Throughout this manuscript, we have assumed that the rate of adaptive substitutions, Λ, and the variance in log fitness, v, are roughly constant over time. However, in both natural and experimental populations, both quantities may fluctuate substantially over time. The key assumption of our analysis, that alleles primarily cause interference at intermediate frequencies but are primarily affected by interference at very low frequencies, implies that fixation probability $\bar{P}$ should be anti-correlated on the time scale $t^* \sim \log(Ns)/s$ over which alleles go from a few copies to intermediate frequency; in other words, a period of high $\bar{P}$ will create an excess of interference-causing alleles $\sim t^*$ generations later, lowering $\bar{P}$ at that time, and conversely for periods of low $\bar{P}$. This effect can clearly be seen in our simulations (Figure S9), which began with monomorphic populations (as in many microbial evolution experiments), so that initially interference was low and $\bar{P}$ was high.
