# Supplementary material for: Limits to the Rate of Adaptive Substitution in Sexual Populations
Source: PLoS Genet. 2012 Jun 7;8(6):e1002740. doi: 10.1371/journal.pgen.1002740 (PMC3369949; doi:10.1371/journal.pgen.1002740)
Supplement: Text S6 — Variation among backgrounds and spatial variation. (PDF) [file pgen.1002740.s016.pdf]

Text S6 Variation among backgrounds and spatial variation

Whitlock [58] has developed a simple approximation for the fixation probability in a spatially subdivided population: $\bar{P} = 2s\,(N_e/N)\,(1 - F_{ST})$, where N_e is the long-term effective size of the whole population, and F_{ST} is the standardized variance of neutral allele frequency in that population. This bears some resemblance to our general expression for fixation probability, $\bar{P} = 2s/[V(1 + \mathrm{C.V.})]$ (from Eq. (3)): both expressions involve a measure of variation between demes or genetic backgrounds, and both show that this variation necessarily reduces fixation probability. However, there are also several crucial differences. F_{ST} measures the variance in neutral allele frequency, whereas C.V. is the coefficient of variation of fixation probability between demes or genetic backgrounds. [58]'s N_e measures the long-term loss of variation and is equivalent to our Υ, whereas our V measures the short-term rate of drift. The derivations are also quite different. [58] makes the approximation that there is a separation of timescales, such that the overall allele frequency, $\bar{p}$, diffuses with mean change $M = s\,(1 - F_{ST})$, and variance $\bar{p}\bar{q}/\,(2N_e)$. In contrast, we average over the set of coupled equations for fixation probability $P\left(\underline{X}\right)$ in each location or genetic background, $\underline{X}$. We have shown that Υ (and therefore [58]'s N_e) may be greatly reduced while $\bar{P}$ (i.e., C.V. and V) is not much affected, and, conversely, that $\bar{P}$ may decrease while Υ remains nearly constant (Figure 6). It might still be the case that [58]'s approximation applies to our model, if F_{ST} is sufficiently large for cases where $C.V.$ is large. However, we do not see how to show this, by calculating F_{ST} in a general way. Moreover, our results for a linear map apply when recombination and selection act at similar rates, so that the separation of timescales required by Whitlock's derivation does not apply.
